# A dual regulation mechanism of histidine kinase CheA identified by combining network-dynamics modeling and system-level input-output data

**DOI:** 10.1371/journal.pcbi.1006305

**Published:** 2018-07-02

**Authors:** Bernardo A. Mello, Wenlin Pan, Gerald L. Hazelbauer, Yuhai Tu

**Affiliations:** 1 IBM T. J. Watson Research Center, Yorktown Heights, New York, United States of America; 2 Physics Institute - University of Brasilia, Brasilia, Brazil; 3 Department of Biochemistry, University of Missouri, Columbia, Missouri, United States of America; University of Illinois at Urbana-Champaign, UNITED STATES

## Abstract

It is challenging to decipher molecular mechanisms in biological systems from system-level input-output data, especially for complex processes that involve interactions among multiple components. We addressed this general problem for the bacterial histidine kinase CheA, the activity of which is regulated in chemotaxis signaling complexes by bacterial chemoreceptors. We developed a general network model to describe the dynamics of the system, treating the receptor complex with coupling protein CheW and the P3P4P5 domains of kinase CheA as a regulated enzyme with two substrates, ATP and P1, the phosphoryl-accepting domain of CheA. Our simple network model allowed us to search hypothesis space systematically. For different and progressively more complex regulation schemes, we fit our models to a large set of input-output data with the aim of identifying the simplest possible regulation mechanisms consistent with the data. Our modeling and analysis revealed novel dual regulation mechanisms in which receptor activity regulated ATP binding plus one other process, either P1 binding or phosphoryl transfer between P1 and ATP. Strikingly, in our models receptor control affected the kinetic rate constants of substrate association and dissociation equally and thus did not alter the respective equilibrium constants. We suggest experiments that could distinguish between the two dual-regulation mechanisms. This systems-biology approach of combining modeling and a large input-output dataset should be applicable for studying other complex biological processes.

## Author summary

In complex biological systems, it is often difficult to determine which steps in the underlying biochemical network are regulated by the signal by using direct experimental measurements alone. In this paper, we tackled this general problem in the case of the kinase activity of the multi-domain histidine kinase CheA. We developed a quantitative reaction network model to describe the CheA enzyme kinetics by considering all the key reaction steps explicitly. We used this general model with different regulation schemes of progressively increasing complexities to fit a large input-output dataset. Our modeling revealed novel dual regulation mechanisms in which receptor activity regulated two independent reactions in the network including the ATP binding reaction that was previously unsuspected. Through our quantitative analysis, we found that receptors affected the kinetic rate constants of substrate association and dissociation equally and thus did not alter the respective equilibrium constants. Testable predictions of the kinase activity dynamics are made from our models to further distinguish the different dual regulation mechanisms. Our study shows that combining modeling kinetics of the reaction network and input-output data can help reveal the underlying regulation mechanism in complex networks where probing individual reaction is impossible.

## Introduction

Living systems sense environmental signals and respond by altered behaviors. Control of behavior is achieved by a myriad of biochemical reactions forming a reaction network,in which some reactions are directly regulated by the sensed signals. However, exactly which reactions are controlled by a signal and how that control is exerted are incompletely understood. Experimentally, it is common that only the final outputs can be measured, whereas changes in internal processes remain largely inaccessible. Given these limitations in our knowledge of the network and in the available data, inferring the underlying regulation mechanisms from systems-level input-output measurements is a major challenge in systems biology, especially for complex reaction networks [1]. Here, we address this challenge in understanding regulation mechanisms for the specific case of histidine kinase CheA which is a central component in the machinery of bacterial chemotaxis and which is regulated by bacterial chemoreceptors [2–4].

The first stage of signal transduction in bacterial chemotaxis is from the external stimulus (a chemical) to a conformational change in the transmembrane chemoreceptors [5–7]. The second stage is from the conformation state of the receptors to modulation of phosphorylation of the response regulator CheY [8, 9]. The final stage is control by phosphorylated CheY of the bacterial rotary motor and thus the pattern of cell movement [10]. The second stage involves histidine kinase CheA, which catalyzes phosphoryl transfer between ATP and a histidinyl side chain in its P1 domain [11, 12]. That phosphoryl group is then transferred to an aspartyl residue on CheY. Phosphoryl transfer from ATP to His to Asp is the hallmark of two-component regulatory systems, which are prevalent in microorganisms [13].

Phosphoryl transfer from ATP to CheY involves steps internal to CheA [14]. These are chemical reactions involving ATP, CheY, and three domains of CheA: P1 (phosphoryl-accepting), P2 (CheY-binding), and P3P4P5 (dimerization, catalytic and receptor-coupling, respectively) [15, 16]. These components define chemical states connected by bi-directional (forward and backward) reactions, forming a reaction network.

The P3P4P5 unit plays a central role in the network, as the enzyme that catalyzes phosphoryl transfer between ATP and P1. For enzymatic reactions with one enzyme [*E*]_tot_ and one varied substrate [*S*], experimentally measured reaction rates are typically fit to the Michaelis-Menten equation,

$$v=\frac{k_{\text{cat}}^{S}{\left[E\right]}_{\text{tot}}\left[S\right]}{K_{m}^{S}+\left[S\right]}.$$ (1)

For chemotaxis phosphoryl transfer, this straightforward analysis has been used to determine how different receptor states regulate the Michaelis-Menten constant $K_{m}^{S}$ and the catalytic rate constant $k_{\text{cat}}^{S}$ [17]. However, such analysis cannot reveal underlying regulatory mechanisms [18].

To determine which steps of the reaction network are regulated and how they are regulated is essential to understand the signaling mechanism. However, it is also an extremely challenging task because it is difficult, if not impossible, to probe experimentally each individual reaction in the network separately. In this paper, we investigate the regulatory mechanism of kinase CheA by modeling the kinetics of the entire enzymatic reaction network using different hypotheses of regulation. Models were fit to an entire set of experimental data with the aim of determining which hypotheses were consistent with the data. The best-fitting models suggested experiments to distinguish among them. This systematic approach, demonstrated here for CheA regulation, should be applicable to investigations of other complex biochemical networks.

## Models and methods

Our modeling utilized primary data generated by experiments described in Pan et al. [17]. Those studies characterized kinase activity of functional chemotaxis signaling complexes formed by the P3P4P5 units of CheA, chemoreceptor Tar and the adaptor protein CheW.

The experiments consisted of changing the concentration of one of the two substrates (ATP or the isolated CheA phosphoryl-accepting domain P1) while holding constant the concentration of the other substrate, for different signaling states of the chemotaxis signaling complex. These states were generated by the extent of receptor modification at the four methyl-accepting sites, which changes receptor signaling state from all-glutamyl (Tar-4E) kinase-off to all-glutaminyl (Tar-4Q) kinase-on, or by increasing the concentration of the attractant ligand aspartate, which shifts the signaling state toward kinase-off. Chemoreceptors were rendered functional by insertion into native *E. coli* phospholipid bilayers provided by nanodiscs or native *E. coli* membrane vesicles. From the phosphorylated P1 (P1P) concentration accumulated at time Δ*t*, shortly after the reaction was initiated, the average kinase activity ($\bar{v}$ = average reaction velocity) and the average kinase activity per enzyme molecule ($\bar{k}$ = the reaction velocity per enzyme molecule, i.e. the apparent catalytic rate constant) were computed as

$$\bar{v}=\frac{\left[\text{P1P}\right]}{\Delta t},\;\;\;\bar{k}=\frac{\bar{v}}{\left[P3P4P5\right]}.$$ (2)

The experiments generated 20 “input-output” curves of $\bar{k}$ versus [*P*1] or [*ATP*] (two response curves for each of the 10 receptor states). The Michaelis-Menten equation was used in [17] to describe each experimental curve separately. Each curve required a pair of parameters $k_{\text{cat}}^{S}$ and $K_{m}^{S}$, and thus 40 parameters were used to fit all the data without any necessary connections among these 40 phenomenological parameters. While CheA kinetics with isolated subunits have been studied before [15, 16, 19], the recent studies [17] were specially suitable for investigating possible receptor regulation mechanisms using our modeling approach because of the large number of receptor states investigated.

### A network model for CheA enzymatic reaction dynamics

**Fig 1:**
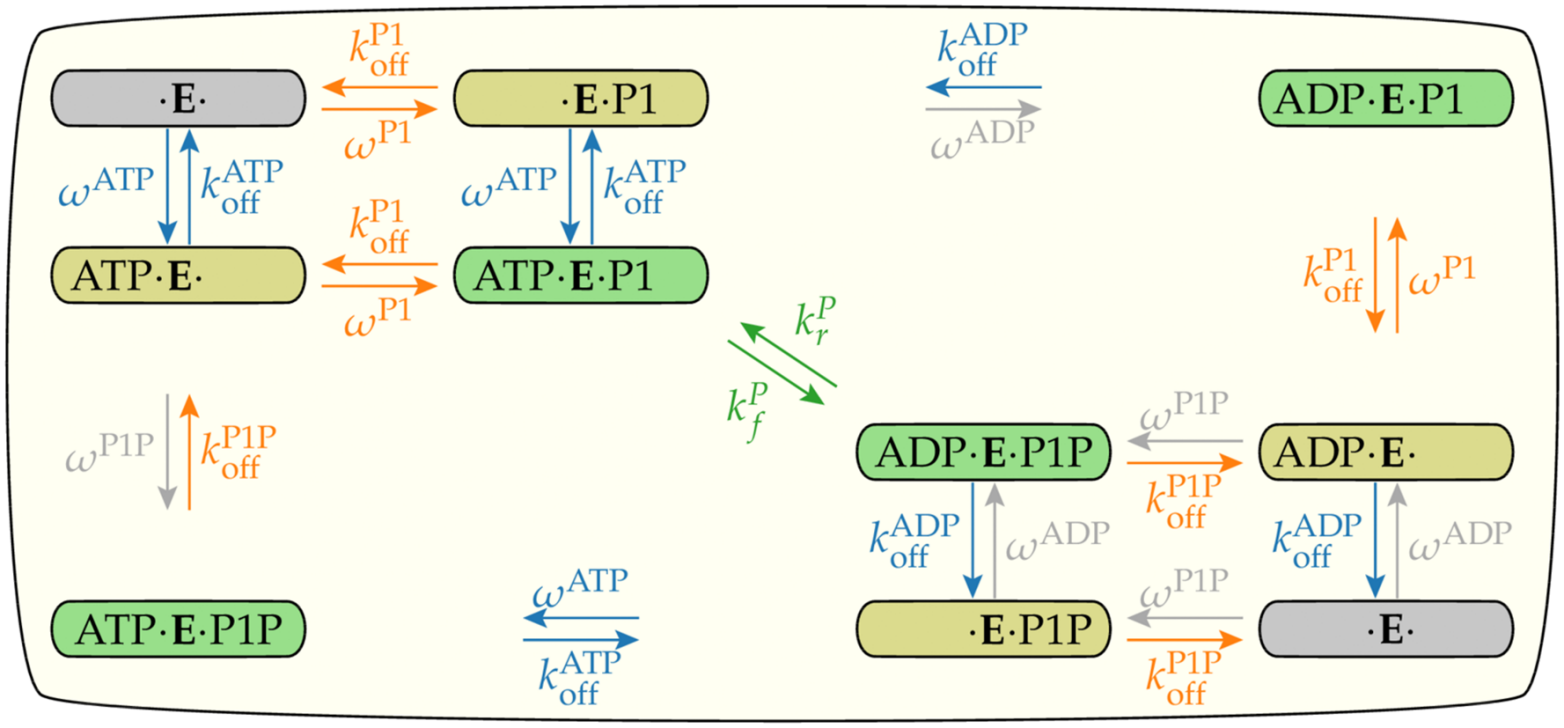
The enzymatic reaction network. The enzyme P3P4P5, denoted by *E*, catalyzes the phosphoryl transfer between its two substrates ATP/ADP and P1/P1P. There are nine states related to the binding of ADP, ATP, P1 or P1P to the enzyme (the empty state is drawn twice). Each pair of vertical or horizontal arrows indicates the association and dissociation of one substrate to the enzyme. The diagonal arrows in the middle indicate the phosphoryl transfer reactions. For each substrate *S*(= *ATP*, *P*1), $k_{\text{off}}^{\text{S}}$ is the dissociation rate constant and *ω*^*S*^ is described by Eq 3 and is proportional to the association rate constant. The gray arrows and rate constants indicate reactions that are negligible because of the low levels of ADP and P1P in the experiments. Reactions that belong to the same regulated mechanism are drawn with the same color, blue for ATP/ADP association/dissociation, orange for P1/P1P association/dissociation, and green for phosphoryl transfer. The unit of all rates constants is s^−1^.

To explain this full set of data within a coherent framework, we developed a simple enzymatic network model to describe dynamics of the enzyme in all possible states in combination with its two substrates/products (ATP/ADP, P1/P1P). The enzyme has two binding sites, one for ATP or ADP and other for P1 or P1P. Each binding site can be in three states: empty, occupied by substrate or by product. The combination of these occupancy states results in 3 × 3 = 9 enzyme configurations shown in Fig 1. The transitions from one state to another form the enzymatic reaction network.

The association/dissociation dynamics of substrate *S*(= *ATP*, *P*1) to the enzyme are described by the dissociation rate constant, $k_{\text{off}}^{\text{S}}$, and by the equilibrium dissociation constant, $K_{d}^{S}$, which identify the equilibrium properties of the binding process. For convenience, we defined the *on* rate as

$$\omega^{S}=k_{\text{off}}^{\text{S}}\frac{\left[S\right]}{K_{d}^{S}}.$$ (3)

The experimental design in which the reactions proceeded for only a brief time before sampling allowed us to assume that [ATP] and [P1] were constant and that [*ADP*] ≈ [*P*1*P*] ≈ 0. This latter assumption leads to *ω*^ADP^ ≈ *ω*^P1P^ ≈ 0, represented by gray colored arrows in Fig 1.

The phosphoryl group transfer rate constant for transfer from ATP to P1 is $k_{f}^{P}$. The reverse rate constant $k_{r}^{P}$ describes the opposite transition. The ratio between these two rate constants

$$G^{P}=\frac{k_{r}^{P}}{k_{f}^{P}}=\frac{\left[\text{ATP}\;\cdot \;E\;\cdot \text{P}1\right]}{\left[\text{ADP}\;\cdot \;E\;\cdot \;\text{P}1\text{P}\right]}{|}_{\text{Isol}.\;\text{eq}.}$$ (4)

defines the isolated equilibrium between the two states ([ATP · **E** · P1] and [ADP · **E** · P1P]). The equilibrium properties depend only on the difference of free energy between the two enzyme configurations, which is given by *k*_*B*_*T* ln *G*^*P*^ with *k*_*B*_*T* the thermal energy unit.

Details of the mathematical formulation of the enzymatic dynamics illustrated in Fig 1 are given in the S1 Appendix.

### Modeling enzyme regulations in the network

Besides defining the chemical reaction network that connects different states of the enzyme, another important ingredient of the model was to specify which reactions (links in the network) are regulated by the receptor and how they are regulated. Let us characterize the strength of the receptor’s regulatory activity by 0 ≤ *σ* ≤ 1, *σ* = 0, 1 correspond to the minimum and the maximum activities respectively.

In our network model of the enzyme kinetics, there are three possible steps that the receptor activity (*σ*) could affect: association/dissociation of ATP/ADP, association/dissociation of P1/P1P, and the phosphoryl transfer between ATP and P1. Regulations of different reactions are labeled by different colors of the reaction arrows in Fig 1. In this paper, we consider all three possibilities and their combinations to identify what are the minimal sets of regulations needed to explain all the experimental data [17].

The exact nature of the regulation determines how *σ* affects the reaction rate *k*_*n*_ for the *n*^th^ reaction. Here, we consider the simple case in which the enzyme has two conformations (active and inactive) and the receptor controls the fraction of time the enzyme spends in each conformation. We further assume switching between the two enzyme conformations happens at a timescale much faster than other chemical reactions. As a result, the total reaction rates are weighted averages of the “bare” rates in each enzyme state and can be expressed as simple functions of *σ* depending on the nature of the regulation. Specifically, *σ* dependence is linear if the receptor only affects the kinetic rate constants without changing equilibrium properties of the enzyme, but it is a linear rational function if the receptor also changes the equilibrium properties of the enzyme such as the binding energies (see S2 Appendix). We used our model together with the input-output data to determine the nature of the regulation.

### The error function and the biochemical meanings of the fitting parameters

For each specific model using a specific hypothesis *H*_*i*_, we find the values of the biochemical parameters $\vec{p}$ and the receptor activities $\vec{\sigma }$ that minimize the mean error function *χ*^2^ over all experiments defined as:

$$\chi^{2}\left(H_{i}\right)=\min_{\vec{p},\vec{\sigma }}\frac{1}{N}\sum_{j=1}^{10}N_{j}\chi_{j}^{2}\left(\vec{p},\sigma_{j}|H_{i}\right),$$ (5)

where *j* ∈ [1, 10] labeled all the 10 individual receptor states characterized by the membrane preparation (“v” for vesicle, “d” for nanodisc), receptor methylation level (EEEE, QEQE, QQQQ), and the ligand (aspartate) concentration (in *μM*). The total number of data points was $N=\sum_{j=1}^{10}N_{j}$, where *N*_*j*_ was the number of experimental points in each state (between 12–14 data points). The error (loss) function $\chi_{j}^{2}$ for experiment *j* was defined as

$$\chi_{j}^{2}\equiv \frac{1}{{\text{max}}_{j}\left(y\right)}\sum_{\begin{array}{c} i\;\;\text{in}\\ \text{state}\;\;j \end{array}}\frac{{\left(y_{i}-f\left({\vec{x}}_{i}\right)\right)}^{2}}{y_{i}},$$ (6)

where the difference (residue) between the data value and the model fit, $\left(y_{i}-f\left({\vec{x}}_{i}\right)\right)$, was scaled by the geometric mean of the experimental value *y*_*i*_ and the maximum value max_*j*_(*y*) of the entire experiment *j*. We used this definition of error function to avoid giving too much weights to data points with large measured values.

All model parameters fell into two categories. The first category of parameters $\vec{p}$ included three kinetic rate constants ($k_{\text{off}}^{\text{ATP}}$, $k_{\text{off}}^{\text{P1}}$, $k_{f}^{P}$) and three equilibrium constants ($K_{d}^{\text{ATP}}$, $K_{d}^{\text{P1}}$, *G*^*P*^), which described the basic chemical reactions: association/dissociation of P1 and ATP to the enzyme and phosphoryl transfer between bound P1 and ATP. These “bare” biochemical parameters are the same in all experiments. The second category of parameters was the experiment-specific receptor activities *σ*_*j*_, which could modulate the biochemical rates for each different receptor state *j*(∈[1, 10]). We set the activity of the most active receptor to be unity (*σ*_10_ = 1) to set the scale of the receptor activities, and the number of activity parameters was reduced to 9. Thus there are 15 parameters in our model, which is used to fit all the 20 experimental response curves simultaneously. We used a non-linear solver (Levenberg-Marquardt algorithm of the function *curve_fit* from the package *SciPy* [20]) to find the set of parameters ${\vec{p}}^{*}$ and ${\vec{\sigma }}^{*}$ that minimizes the mean error *χ*^2^ defined by Eq 5.

### The parameter sensitivity analysis

For statistics based data fitting where the underlying mechanism is unknown, the balance between improvement in the fitting and the number of parameters may be assessed by information criteria such as the Bayesian [21] or Akaike [22] methods. Here, the structure of our model and the model parameters were all based on known underlying biochemical processes. Therefore, the number of parameters *N*_*total*_ in our models does not vary widely as shown in Table 1 and *N*_*total*_ can not be used effectively to differentiate different models/hypotheses. Instead, by following the approach used in the *Global Kinetic Explorer* [23], we studied the sensitivity of the error function *χ*^2^ with respect to changes in each model parameter and the correlations between different parameters.

**Table 1:** Descriptions of different models based on different regulation hypotheses. Each numbered row corresponds to a particular model (hypothesis). The second column shows the parameters that are regulated by the signal *σ*. The fitted parameters shown in the third column are the parameters varied to minimize the mean error *χ*^2^, Eq (5), whose values are given in the last column. The *K*_*p*/*D*_ parameter is present when a different dissociation constant is used for nanodiscs from that used for the vesicles. The *k*_off/0_ is the residue activity level of the “inactive” state. The fourth column shows the fixed large rate constants for the fast reactions in the corresponding model (see text for explanation). *N*_*total*_ is the total number of fitting parameters; it is the sum of the number of parameters in the second column plus the 12 parameters common to all models shown in the bottom row. The definitions of the symbols used in this table can be found in Table A of the S1 Appendix.

Models (Hypotheses)	Parameters				
	Regulated	Fitted	Fixed	*N*_total_	*χ*^2^
*H*_1_	$k_{\text{off}}^{\text{P1}}$	$k_{\text{off}}^{\text{ATP}},k_{\text{off}}^{\text{P1}},k_{f}^{P}$		15	0.138
*H*_2_	$k_{f}^{P}$	$k_{\text{off}}^{\text{P1}},k_{f}^{P}$	$k_{\text{off}}^{\text{ATP}}=100\;s^{-1}$	14	0.129
*H*_3_	$k_{\text{off}}^{\text{ATP}}$	$k_{\text{off}}^{\text{ATP}},k_{\text{off}}^{\text{P1}}$	$k_{f}^{P}=100\;s^{-1}$	14	0.106
*H*_4_	$k_{\text{off}}^{\text{ATP}},k_{f}^{P}$	$k_{\text{off}}^{\text{ATP}},k_{f}^{P}$	$k_{\text{off}}^{\text{P1}}=100\;s^{-1}$	14	0.092
*H*_5_	$k_{\text{off}}^{\text{ATP}},k_{\text{off}}^{\text{P1}}$	$k_{\text{off}}^{\text{ATP}},k_{\text{off}}^{\text{P1}},k_{f}^{P}$		15	0.089
*H*_6_	$k_{\text{off}}^{\text{ATP}},k_{f}^{P}$	$K_{d/D}^{\text{P1}},k_{\text{off}}^{\text{ATP}},k_{f}^{P}$	$k_{\text{off}}^{\text{P1}}=100\;s^{-1}$	15	0.086
*H*_7_	$k_{\text{off}}^{\text{ATP}},k_{\text{off}}^{\text{P1}}$	$K_{d/D}^{\text{P1}},k_{\text{off}}^{\text{ATP}},k_{f}^{P},k_{\text{off}}^{\text{P1}}$		16	0.083
*H*_8_	$k_{\text{off}}^{\text{ATP}},k_{\text{off}}^{\text{P1}}$	$K_{d/D}^{\text{P1}},k_{\text{off}}^{\text{ATP}},k_{\text{off}/0}^{\text{ATP}},k_{f}^{P},k_{\text{off}}^{\text{P1}}$		17	0.082
Included in all:		$G^{P},K_{d}^{\text{ATP}},K_{d}^{\text{P1}},\sigma_{1},\cdots ,\sigma_{9}$	*σ*_10_ = 1		

The error function landscape in the parameter space was investigated by fixing a particular parameter *p*_*i*_ to a value around its optimal value $p_{i}=x_{i}p_{i}^{*}$ and minimizing *χ*^2^ by varying all other parameters *p*_*k*, *k* ≠ *i*_ and all activities $\vec{\sigma }$:

$${\tilde{\chi }}^{2}\left(x_{i}\right)=\min_{p_{k,k\neq i},\vec{\sigma }}\frac{1}{N}\sum_{j=1}^{10}N_{j}\chi_{j}^{2}\left(p_{i}\right).$$ (7)

**Fig 2:**
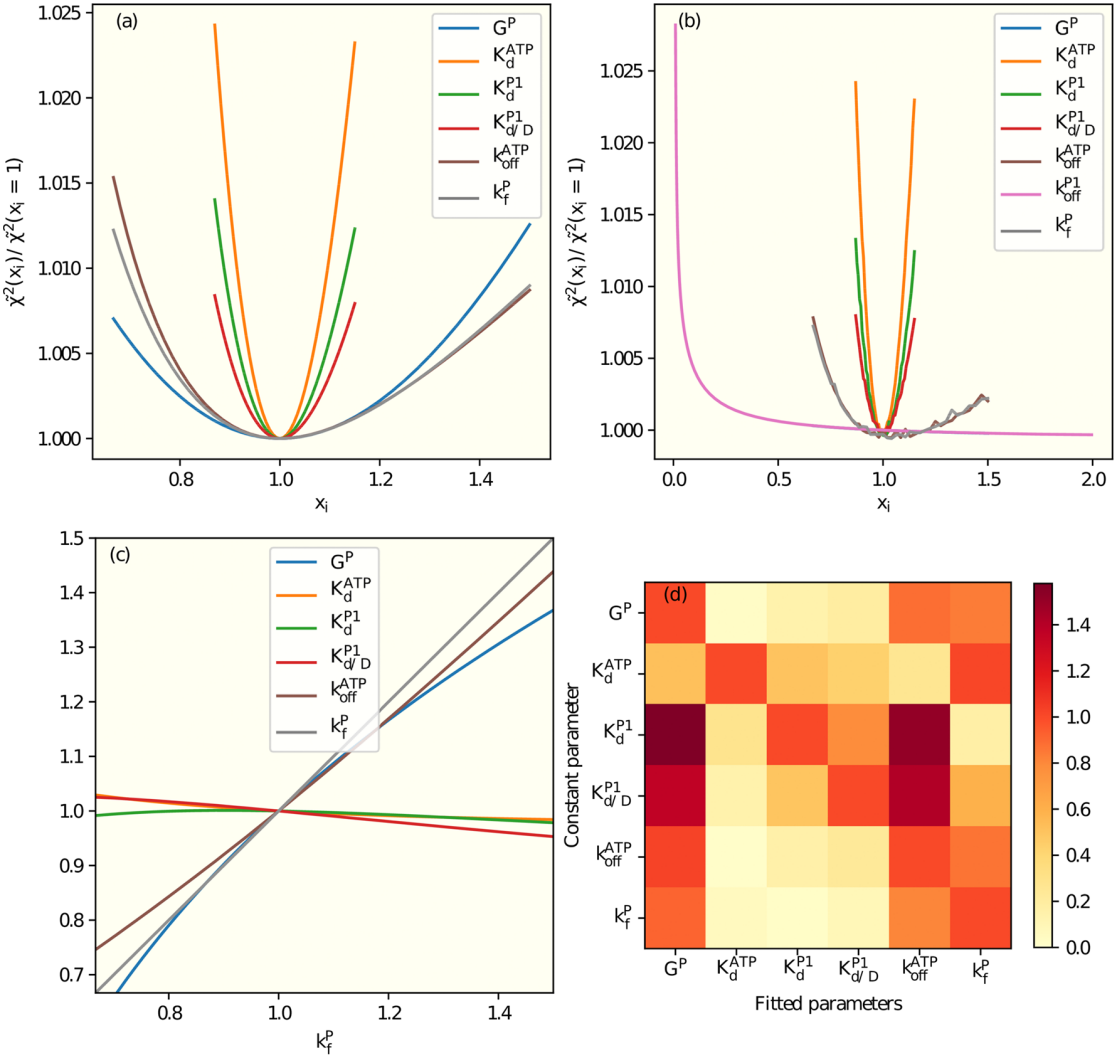
Sensitivity analysis and parameter correlations. (a) Using model 6 (see Table 1), for a given value $x_{i}=p_{i}/p_{i}^{*}$ of the parameter in the legend, the mean error *χ*^2^ is minimized with respect to all other parameters. The resulting minimum ${\tilde{\chi }}^{2}\left(x_{i}\right)$ versus *x*_*i*_ for all individual parameters shown in the legend. (b) Same as (a) except $k_{\text{off}}^{\text{P1}}$ is included as an additional fitting parameter. The dependence of error function on $k_{\text{off}}^{\text{P1}}$ is weak as long as $k_{\text{off}}^{\text{P1}}$ is large enough. This underconstrained problem, which causes the irregularities visible in some curves, is resolved by fixing $k_{\text{off}}^{\text{P1}}=100s^{-1}$ in model 6. (c) When we minimize *χ*^2^ with the parameter $k_{f}^{P}$ fixed at the value shown in the horizontal axis, the other parameters assume values that depend on $k_{f}^{P}$ as shown here. The absolute value of the slope of the dependence, *S*_*ji*_, between the fitted parameter *j* and the fixed parameter *i* characterizes the correlation between the two parameters. (d) *S*_*ji*_ (see (c)) for all pairs of parameters in model 6 are shown in the correlation matrix with the fixed parameters in the columns and the fitted parameters in the rows. The slopes in (c) are represented as the last row in (d).

The dependence of ${\tilde{\chi }}^{2}\left(x_{i}\right)$ on all individual parameters *x*_*i*_ in model 6 were shown in Fig 2(a), where all the curves have the same minimum at *x*_*i*_ = 1. The larger the curvature at the minimum, the more sensitive is the fitting to the change of that parameter. From the second order derivative at the minimum, we can determine a range for the fitted parameter as given in Tables 2 and 3.

**Table 2:** Parameters in the $\left(k_{\text{off}}^{\text{ATP}},k_{\text{off}}^{\text{P1}}\right)$ dual regulation mechanism, model *H*_7_ of Table 1. The fitting is shown in Fig 4. Between brackets are intervals of the reaction rate constants for a 1% increase in *χ*^2^.

$k_{\text{off}}^{\text{P1}}$	318 [156, 1340] s^−1^⋅*σ*	$K_{d}^{\text{P1}}$	195 [175, 219] *μ*M
$k_{\text{off}}^{\text{ATP}}$	20 [11.8, 90] s^−1^⋅*σ*	$K_{d/D}^{\text{P1}}$	310 [270, 359] *μ*M
$k_{f}^{P}$	7.7 [5.8, 11.2] s^−1^	$K_{d}^{\text{ATP}}$	291 [269, 317] *μ*M
*G*^*P*^	9.4 [6.5, 17.0]		

**Table 3:** Receptor’s activity, *σ*, of model *H*_7_ of Table 1. The first letter of the label indicates the membrane preparation: nano*d*isc or *v*esicle. The following four letters show the receptor’s methylation level.

State		[Asp]	*σ*	State		[Asp]	*σ*
dQEQE	1	20 *μ*M	0.0033	vQEQE	6	100 *μ*M	0.0059
	2	5 *μ*M	0.066		7	5 *μ*M	0.035
	3	0 *μ*M	0.31		8	0 *μ*M	0.18
vEEEE	4	10 *μ*M	0.00012	vQQQQ	9	1000 *μ*M	0.0089
	5	0 *μ*M	0.00081		10	0 *μ*M	1

The sensitivity analysis was essential in identifying underconstrained parameters. In particular, when there is a fast reaction with a timescale that is much shorter than the experiment time and the other relevant timescales in the system, the exact value of the rate constant for this ultra fast reaction can not be determined uniquely, only a lower bound can be established. For example, in our model *H*_6_ (see Table 1), if $k_{\text{off}}^{\text{P1}}$ were (unknowingly) treated as a fitting parameter, the sensitivity analysis showed only a weak dependence on $k_{\text{off}}^{\text{P1}}$ as long as it is bigger than a certain value as shown in Fig 2(b). This excessive degree of freedom also impairs the fitting algorithm, which generates the irregularities seen in Fig 2(b). To avoid fitting an underconstrained parameter, we fixed $k_{\text{off}}^{\text{P1}}$ to be a large rate constant $k_{\text{off}}^{\text{P1}}=100s^{-1}$ in model *H*_6_.

The parameters used in our models came from the underlying biochemical reactions and they were not orthogonal in the error function landscape. As shown in Fig 2(c), when we changed $k_{f}^{P}$ from its optimal value and allowed the other parameters to vary to minimize *χ*^2^, the optimal values of $k_{\text{off}}^{\text{ATP}}$ and $k_{\text{off}}^{\text{P1}}$ also changed roughly proportionally to $k_{f}^{P}$ while other parameters only showed weak dependence on $k_{f}^{P}$. The correlation between two parameters can be characterized by the linear proportionality constant between the two parameters. In Fig 2(d), we show all the pair-wise linear correlation constant in a matrix. These correlations can be understood intuitively. Since $k_{f}^{P}$ and $k_{\text{off}}^{\text{ATP}}$ are slow reactions in model *H*_6_ (see Table A in S4 Appendix), they must change together to preserve the qualitative behavior. An increase in the forward phosphoryl transfer reaction rate constant $k_{f}^{P}$ can be partially compensated by an increase in *G*^*P*^, which enhances the reverse phosphorus transfer reaction. These partial compensation mechanisms also explained the lower precision in these parameters as seen in Fig 2(a) as compared with the parameters $K_{d}^{\text{ATP}}$, $K_{d}^{\text{P1}}$, an $K_{d/D}^{\text{P1}}$ that have exclusive control of certain parts of the network.

Additional parameters were added to the model based on realistic biological considerations, e.g., the values of *K*_*d*_ could be different for nanodiscs and vesicles; or possible regulation hypothesis, e.g., there could be a residual kinase activity in the inactive state. When additional parameters allowed a better fitting to the data, a well defined minimum of *χ*^2^ emerged in the error function analysis. This was the case when we considered different values of *K*_*d*_ for nanodiscs and vesicles (model *H*_8_ in Table 1) as shown in Fig. B in S6 Appendix. The number of parameters and the value of *χ*^2^ obtained from a representative subset of models we studied are shown at Table 1. Among the multitude of models we tried, these are the ones with roughly the lowest *χ*^2^ for each number of parameters, with the exception of *H*_1_. It clearly demonstrates that a model does not necessarily fit the data better just because it has more parameters.

## Results

Possible regulation mechanisms (hypotheses), represented by *H*_*i*_, specify how the receptor activity *σ* affected different reactions in the network. For example, hypothesis *H*_1_ was that the receptor activity only affected only P1 binding. For a given hypothesis *H*_*i*_, the total error function was minimized with respect to all parameters and the resulting minimum error *χ*^2^(*H*_*i*_) served as the error of the hypothesis *H*_*i*_.

**Fig 3:**
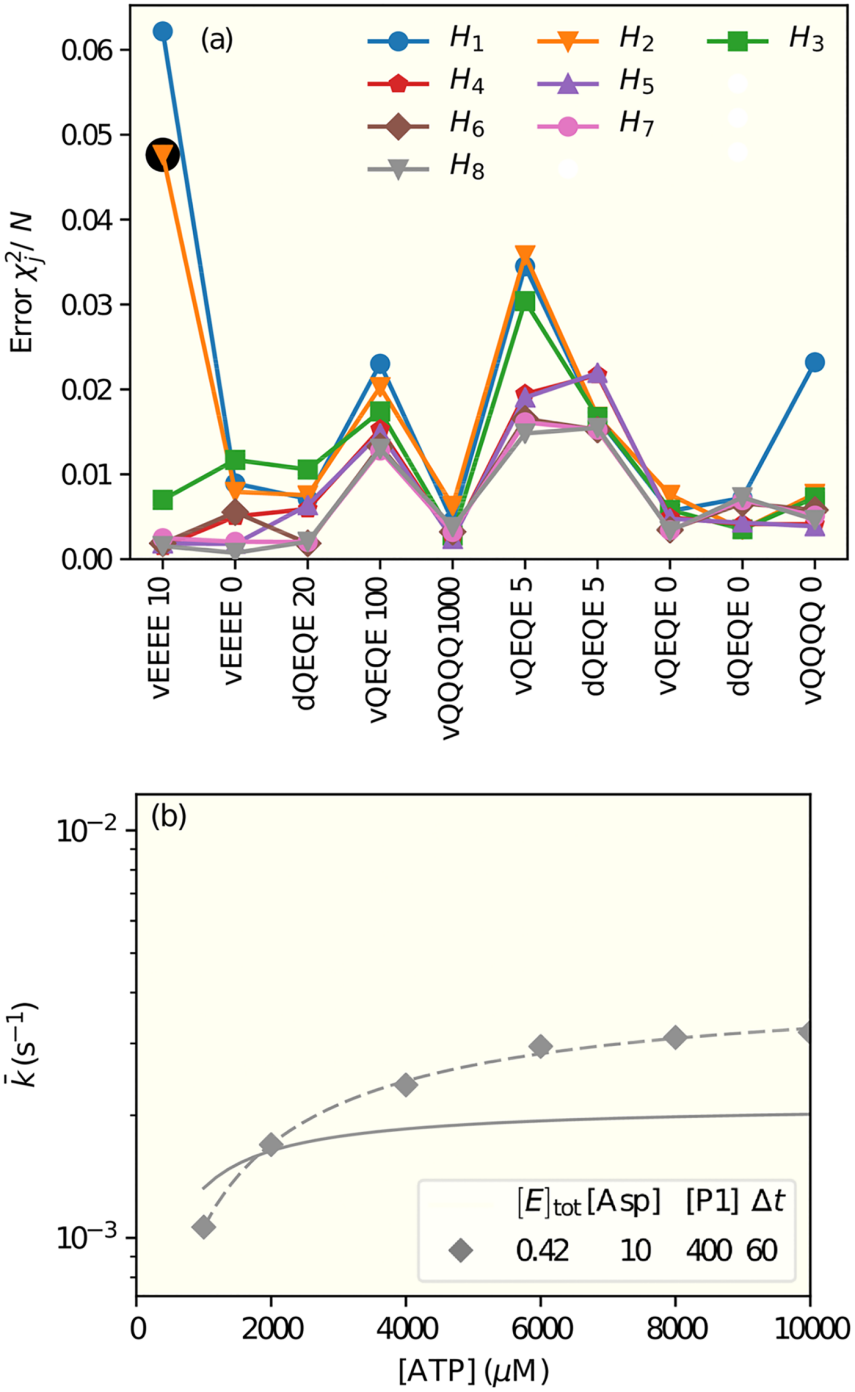
Fitting models to experimental data. (a) Decomposed fitting errors, $\chi_{j}^{2}$, are shown for all 10 experiments. Different color lines (symbols) correspond to models with different hypotheses of regulation, which are described in details in Table 1. The experiments are labeled in the x-axis with the letter ‘d’ for nano*d*iscs or ‘v’ for *v*esicles, four letters for the methylation state, and the concentration of aspartate, in *μ*M. The experiments are arranged in the order of ascending receptor activity (*σ*). The lines connecting the points are only guides to the eyes. (b) The inadequate fit of an unsuccessful model (model *H*_2_ of Table 1) to the data for the least active receptor EEEE in the presence of aspartate that reduces activity to an even lower level. The corresponding error is highlighted by the black circle in (a). The legend shows the concentrations of the enzyme P3P4P5 ([*E*]_tot_), aspartate ([Asp]), and substrate ([ATP] or [P1]), in *μ*M; and the measurement time in seconds. The dashed line is obtained by fitting the data by the Michaelis-Menten equation. This failed model (*H*_2_) in which receptors regulate only the phosphoryl transfer rate $k_{f}^{P}$ generates a lower maximum kinase rate constant and a lower half-maximum ATP concentration in comparison with the data (symbols).

This approach enabled us to look for the regulation mechanism by searching the hypothesis space systematically. Starting from the simplest regulation rules, we looked for significant improvement upon adding new regulatory mechanisms and search for the minimal model(s) that fits all the experimental data. In Fig 3(a), we show the results from some of the tested regulation hypotheses arranged in the legend from the simplest (the top row) to the most complex (the bottom row), see Table 1 for a detailed description of the models. Decomposition of the total error *χ*^2^ into errors for each individual experiments (Fig 3(a)) revealed which experiment(s) invalidated a particular hypothesis and also possible directions for improvement. We describe our main findings below.

### The dual regulation mechanism

As shown in Fig 3(a), none of the three single regulation hypotheses (*H*_1_, *H*_2_, and *H*_3_), i.e., regulating the ATP or P1 binding or the phosphoryl transfer rate constants fit all the experimental data. To our surprise, the single regulation of $k_{\text{off}}^{\text{ATP}}$ (*H*_3_) was much better than the other two single regulation mechanisms. The worst performing single regulation hypothesis was regulating P1 dissociation $k_{\text{off}}^{\text{P1}}$ (model *H*_1_), where the errors from several experiments such as VEEEE10, vQEQE5, vQEQE100, and vQQQQ0 were large. For the model with single regulation of the phosphotransfer rate $k_{f}^{P}$ (model *H*_2_), the fitting of vQQQQ0 improved. However, the error for the vEEEE10 experiment was still large. The reason for the large fitting errors for receptor states like vEEEE10 is due to their lower activities than others. It is the extreme low activity receptor state that shows the largest difference between models with and without certain regulation by receptor activity. The detailed reason for this misfit can be understood by comparing the model results with experimental data directly. As shown in Fig 3(b), the maximum kinase rate and the half-maximum [ATP] concentration for vEEEE10 are both higher in the experiment than in the model. Thus an improvement in fitting this curve seems to suggest an additional regulation of $k_{\text{off}}^{\text{ATP}}$.

Based on results from all single regulation models, we next tried to combine the different regulations. We found that there was a general reduction of errors across most experiments by having $k_{\text{off}}^{\text{ATP}}$ regulation combined with a regulation of either $k_{f}^{P}$ (from *H*_2_ to *H*_4_) or $k_{\text{off}}^{\text{P1}}$ (from *H*_1_ to *H*_5_) without introducing any additional parameters in our model. The decomposed fitting errors for these two successful dual regulation mechanisms are shown in Fig 3(a) (models *H*_4_ and *H*_5_ in the second row of the upper legend). However, the dual regulation of $k_{f}^{P}$ and $k_{\text{off}}^{\text{P1}}$ did not improve the fitting and resulted in a larger error *χ*^2^ = 0.132.

### Receptors mainly regulate the kinetic rate constants not the equilibrium constants

For a given reversible chemical reaction between two states, the receptor activity (*σ*) can change the energy barrier between the two states and thus change the kinetic rate constants by the same factor (linearly proportional to *σ*) without changing their ratio, i.e., the equilibrium constants $K_{d}^{\text{ATP}}$, $K_{d}^{\text{P1}}$, and *G*^*P*^. This was the situation we considered in most of our study. However, we also considered the more general cases in which the receptor activity changed the free energy difference between the two states leading to different dissociation constants for the active (*σ* = 1) and inactive (*σ* = 0) receptors and a more complicated (linear rational function) dependence of the forward and backward rate constants on *σ* (see S2 Appendix). With this new degree of freedom, only slightly improved fittings were achieved as shown in Fig 3(a) (the fourth row in the legend) for a model *H*_8_ with residual activity in P1 binding. We also allowed this new degree of freedom in the single regulation cases, but the fittings did not seem to improve much if at all (see Fig. A in S2 Appendix). Our results suggest that receptors mainly regulate the kinetic rates by controlling the energy barrier between two states without changing their free energy difference.

### Differences between nanodisc- and vesicle- inserted receptors plus other findings

**Fig 4:**
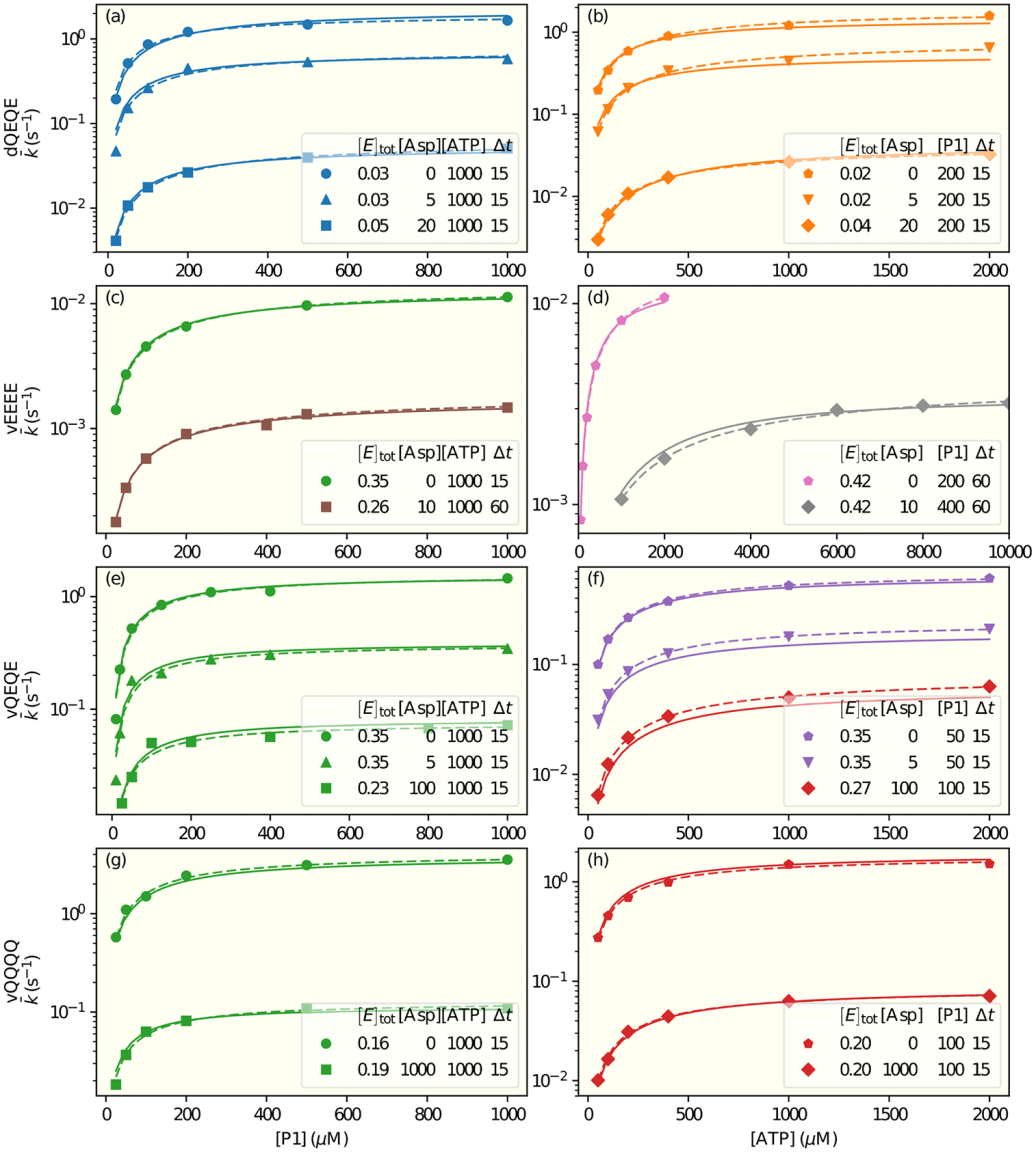
Fits to experimental data. The symbols show apparent enzymatic phosphorylation rate constants from the experiments described in [17]. The data shown in the left panels are from experiments in which [ATP] was kept constant and [P1] varied, and vice versa in the right panels. (a)&(b) show results for receptors in QEQE states in nanodiscs. (c-h) show results for receptors in states EEEE, QEQE, and QQQQ, respectively,in membrane vesicles (v). The legend in each panel shows the concentrations of the enzyme P3P4P5 ([*E*]_tot_), aspartate ([Asp]), and of the constant substrate ([ATP] or [P1]), in *μ*M; and the measurement time in seconds. Dashed lines are fitting each experimental curve independently by the Michaelis-Menten equation. Solid lines are a global fit of all data by our model *H*_7_ with parameters given in Tables 2 and 3.

In both dual-regulation mechanisms, our model indicated that for cases with the same receptor modification state (QEQE) and the same aspartate concentrations (0, 5*μM*), the receptor activities were larger in nanodiscs than in vesicles by ∼60–90% (see Table 3 and Table A in S4 Appendix). The differences could reflect higher activity of receptors removed from the heterogeneous native membrane or a difference between activity of the small clusters of signaling complexes that form when participating receptors are native membrane vesicles [24] and individual core complexes are constructed using receptors inserted in nanodiscs [25]. We also considered the possibilities that there were different values of $k_{\text{off}}^{\text{P1}}$, $K_{d}^{\text{P1}}$, $K_{d}^{\text{ATP}}$, or $k_{\text{off}}^{\text{ATP}}$ for vesicles and nanodiscs. We found that a modest improvement in fitting could be achieved by having different values of $K_{d}^{\text{P1}}$ for nanodisc and vesicles. These hypotheses (*H*_6_ and *H*_7_) are shown in the third row in the legend of Fig 3(a), details of these models can be found in Table 1. Table 2 and Table A in S4 Appendix show the parameters of the two dual-regulation models. In both models (*H*_6_ and *H*_7_), our study suggested a higher value of $K_{d}^{\text{P1}}$ for nanodiscs than that for vesicles by about 50%. The actual fitting of this model (*H*_7_) to the experimental data is given in Fig 4.

We also explored the possibility of the binding of one substrate depending on the presence of the other substrate as investigated in [26, 27] and the possibility of $K_{d}^{S}$ and $k_{\text{off}}^{\text{S}}$ being different for for P1 and P1P or for ATP and ADP as proposed in [28]. However, including these possibilities did not improve the fitting of the available data. Further experiments are needed to explore these more detailed hypotheses.

## Discussion

**Fig 5:**
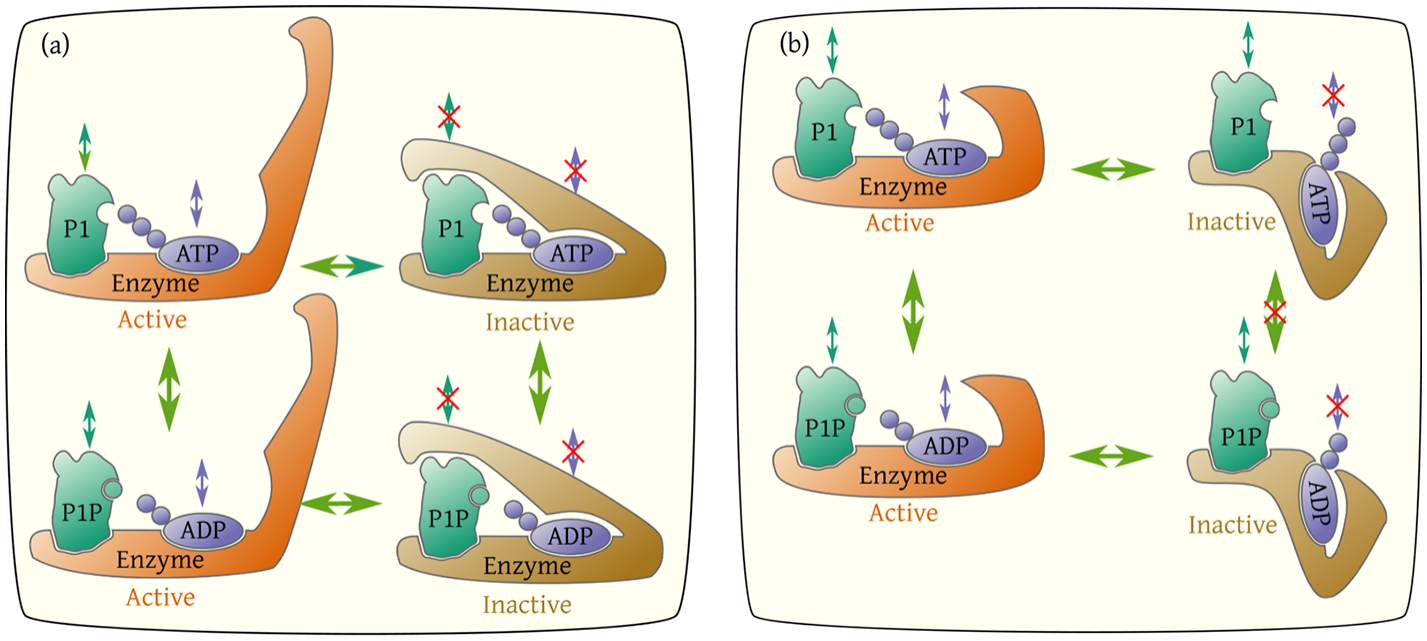
Illustration of the two possible dual regulation mechanisms. The enzyme (P3P4P5) has two states: active and inactive. The receptor activity controls the probability of the enzyme being active. In both mechanisms, the ATP binding site is open in the active state and closed in the inactive state. In addition to regulating ATP binding, the receptor activity also regulates either the P1 binding as shown in (a) or the phosphoryl transfer between ATP and P1 as shown in (b).

In this paper, we developed a simple network model to study the regulatory mechanism of the multi-domain histidine kinase CheA based on an extensive body of kinetic measurements. Our best-fit models identified two possible mechanisms for regulation by receptors. In one, regulatory signal from the receptor controls ATP and ADP association/dissociation rate constants and either P1 association/dissociation rate constants or the phosphoryl transfer rate constants. Previous experimental [27, 28] and numerical studies have already suggested that ATP binding was controlled by receptor activity. However, the dual regulation mechanism identified is new to the best of our knowledge. Furthermore, our study showed that receptors modulate forward and backward rate constants equally by controlling the barrier between the active and inactive states of the enzyme. The two dual regulation mechanisms are illustrated in Fig 5. They are consistent with recent molecular dynamics simulations that identified the existence of two states with one of them blocking access of substrate to its binding site [29, 30].

The full network model of the enzymatic reactions can be simplified by exploiting the separation of time scales in the reactions, detailed in S3 Appendix. These simplifications help us gain more insights into the dynamics of the CheA kinase activity, which will be integrated with the downstream elements (methylesterase CheB, response regulator CheY, and its phosphatase CheZ) to help us understand the entire pattern of chemotaxis signaling dynamics. They also lead to predictions for future experiments to discriminate further among the remaining regulation hypotheses. We describe some of these insights and predictions below.

### Multiple timescales in the CheA enzymatic reaction network

**Fig 6:**
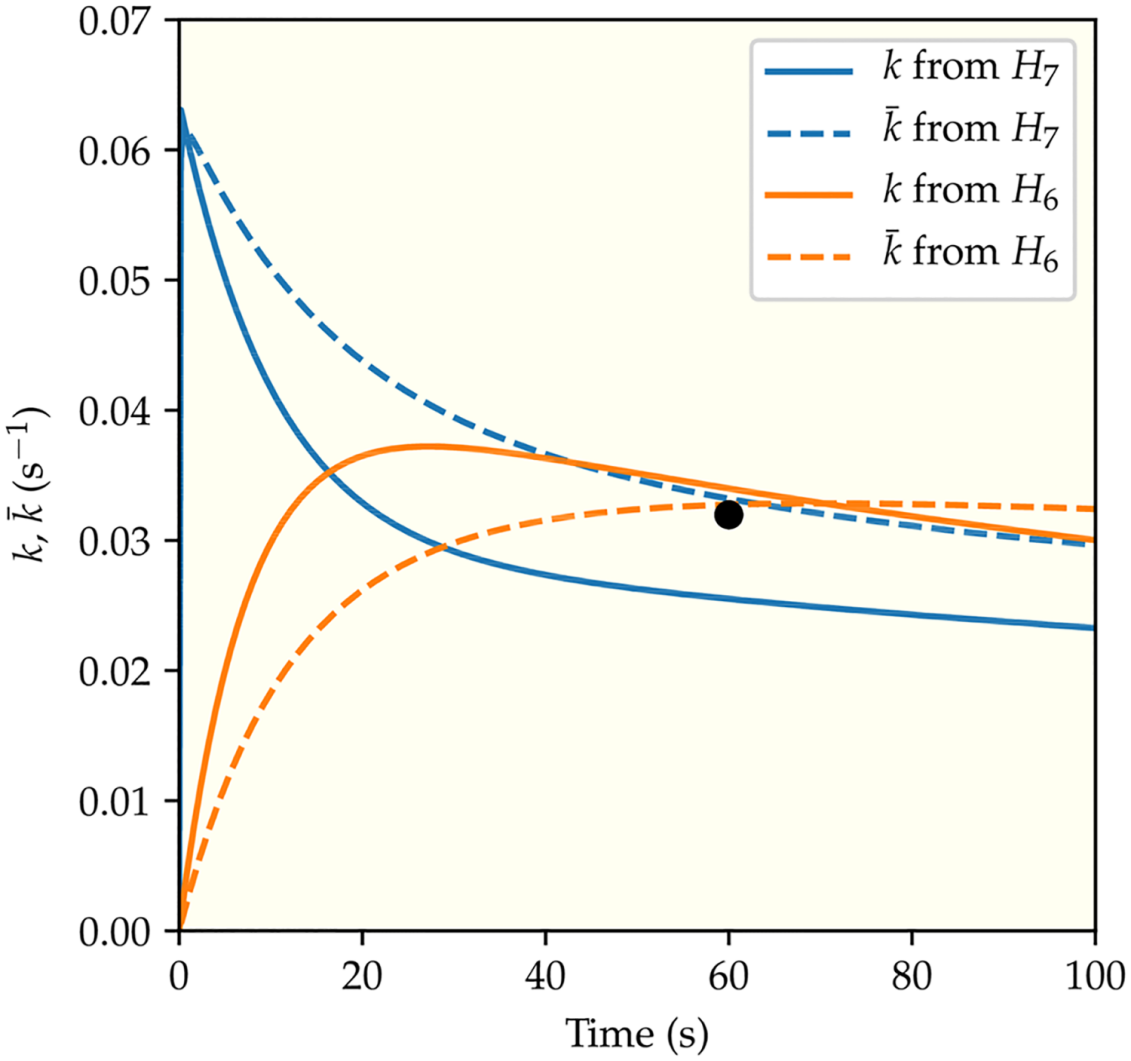
The predicted kinetics. The time dependence of the apparent phosphoryl transfer rate constants for the two different dual-regulation mechanisms (*H*_6_ and *H*_7_) are represented by the two different colors (blue for regulating $k_{\text{off}}^{\text{ATP}}$ and $k_{\text{off}}^{\text{P1}}$ (*H*_7_) and orange for regulating $k_{\text{off}}^{\text{ATP}}$ and $k_{f}^{P}$ (*H*_6_)). The system studied here contains the least active receptors (EEEE) in membrane vesicles with [Asp] = 10 *μ*M, [ATP] = 10^4^ *μ*M, and [P1] = 400 *μ*M, which reproduces the conditions of the rightmost point of the lower curve in Fig 4(d). The solid and the dashed lines correspond to the instantaneous (*k*) and the average ($\bar{k}$) phosphorylation rate constants respectively. The black circle is the experimental data point from [17].

Dynamics of the enzymatic network are determined by the transition rate constants between different states in the network. These different rate constants give rise to different time scales, which can be regulated by receptor activity. To demonstrate the importance of time scales, in Fig 6 we plot the apparent phosphoryl transfer rate constant as a function of time for the least active receptor EEEE. We followed the same procedure as in the experiments that generated the data we have analyzed, i.e. pre-mixing P1 with the enzyme and then adding ATP at time *t* = 0. Depending on details of regulation by receptors, the apparent phosphorylation rate constant could either decrease after an initial fast surge (the blue lines) or increase from zero gradually before converging to its steady state value (the orange lines).

The convergence to the steady state is characterized by the relaxation time, *τ*. Precise calculation of the relaxation time is presented in the SM, but it can be estimated as the inverse of the lowest reaction rate constant. From the inverse of $k_{\text{off}}^{\text{ATP}}$ from Tables 2 and 3, the longest relaxation times were observed for kinase control by EEEE receptors, estimated as 54 s for [Asp] = 0 and 362 s for [Asp] = 10 *μ*M. This means that the average phosphorylation rates determined in the experiments with Δ*t* = 15 s, 60 s are not steady state rates. We have taken this time-dependent effect into account explicitly in all our model fittings.

**Fig 7:**
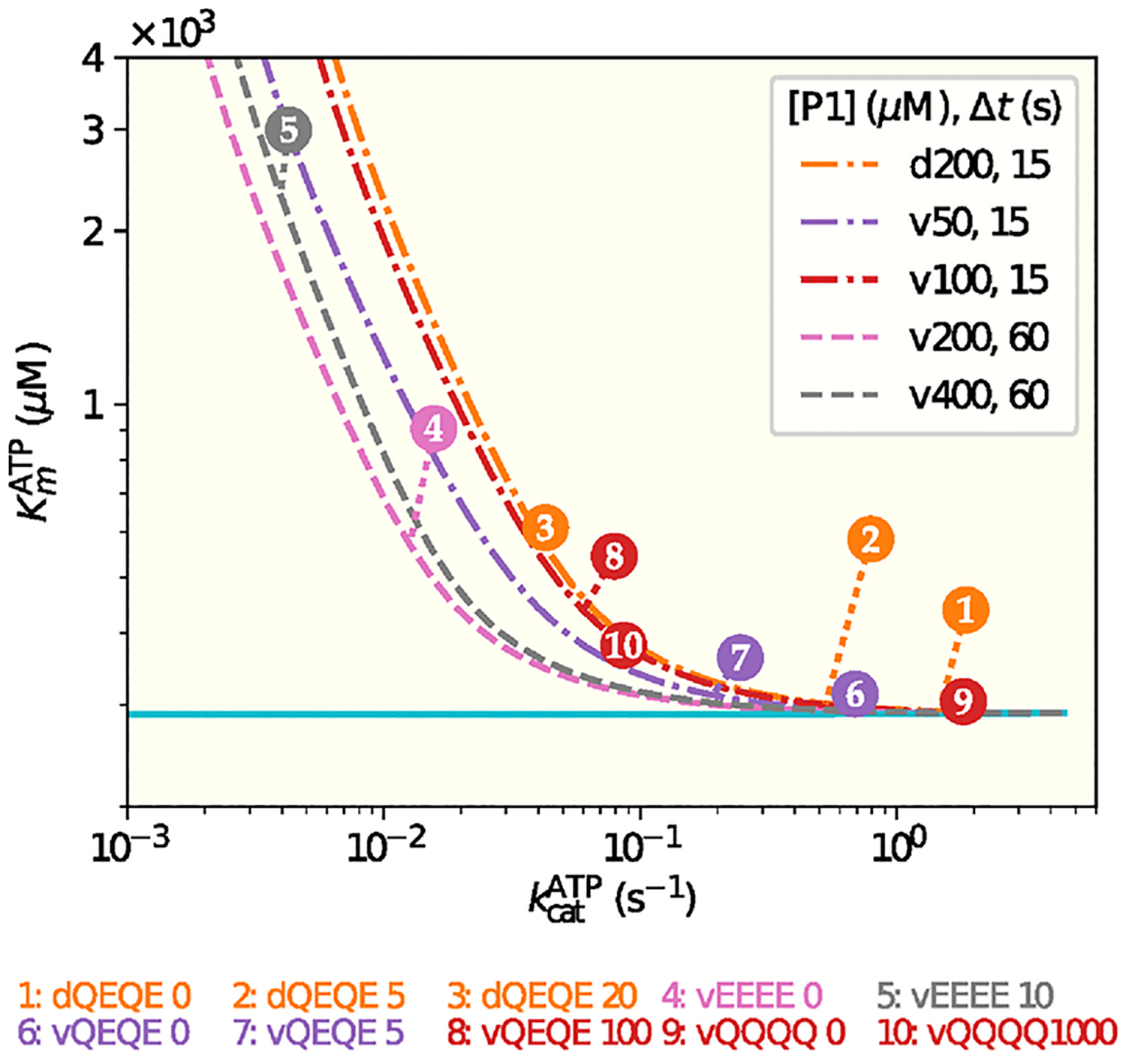
The phenomenological Michaelis-Menten (MM) parameters depend on the receptor activity and measurement time. The pair of the MM parameters ($K_{m}^{j}$,$k_{\text{cat}}^{j}$) for a given experiment *j* ∈ [1, 10] is given by the circle with the experiment number *j*, which is defined the same way as in Fig 3(a). The dashed lines represent the MM parameter pairs obtained from fitting the corresponding model results with different receptor activities *σ* and at the experimental measurement times (15*s* or 60*s*). It is evident that the effective MM parameters depend on the receptor activity and the measurement time Δ*t*. As Δ*t* → ∞ (ploted as a solid line), the effective *K*_*m*_ approaches a constant independent of *σ*. Details on construction of these curves can be found in the Supplementary Material, S5 Appendix.

Since Michaelis-Menten (MM) analysis of enzyme kinetic data assumes a steady state in which the concentration of enzyme-substrate complex is constant and thus substrate binding to the enzyme is equilibrated, if this condition is not met the MM parameters obtained from the analysis will be incorrect. This can be a problem for analysis of very low activity receptors which generate very low kinase catalytic rate constants (*k*_*cat*_). However, the effective (phenomenological) MM fitting parameters provide useful information by identifying maximum reaction rates and thus rate constants, as well as the substrate concentration, *K*_*m*_, at which the reaction rate is half maximum. We determined the values of these effective MM parameters for curves obtained from our model and compared them with those obtained from direct fitting of the experimental data with the MM equation. There was good agreement between MM parameters obtained in the two ways. However, our analysis also illustrated the potential errors in determining in MM parameters in conditions in which reaction rate constants are very low and thus care must be taken to insure that experimental samples are taken after sufficient time for the MM steady-state assumption to be valid (Fig 7). Specifically, long relaxation times for low values of $k_{cat}^{S}$ lead to effective *K*_*m*_ values that depend on the measurement time Δ*t* (see Fig 7), because the MM requirement for a steady state is not met.

### Transient dynamics and testable predictions

The rich dynamics of the enzymatic reaction network before it reaches its steady state suggest future experiments that can be used to distinguish the remaining hypotheses. By using our model, we can determine the experimental conditions in which the different hypotheses lead to different dynamic behaviors. The difference in dynamic behaviors is most prominent for the less active receptors (EEEE) where the relaxation times are long.

Fig 6 illustrates how two of the best fitting models (*H*_6_ and *H*_7_), both of which explain the existing experimental data at a particular time Δ*t* = 60*s*, lead to distinctive phosphorylation time courses due to their different rate limiting steps. Model *H*_8_ is slightly better than *H*_7_, but has an extra parameter that does not significantly alter the dynamics. For the sake of simplicity we will use *H*_7_. In the dual regulation model (*H*_7_) of ATP and P1 binding, both P1 and ATP bindings are the rate limiting steps for low receptor activity states such as the EEEE receptor with a aspartate concentration [*Asp*] = 10 *μM*. In the experiments that generated the data we analyzed, P1 was mixed with the enzyme prior to initiation of the reaction by addition of ATP thus bypassing this limiting step, the other limiting step is lifted by a large ATP concentration [*ATP*] = 10 *mM*. As a result, the phosphorylation rate rises quickly to its maximum before decreasing to its steady state value as shown in Fig 6 (blue lines). In the other dual regulation model (*H*_6_) of ATP binding and phosphoryl transfer, a much slower initial increase in the phosphorylation rate is predicted (orange lines in Fig 6) because the rate limiting phosphoryl transfer reaction can not be bypassed. The full time-dependent phosphorylation rates shown in Fig 6 represent quantitative predictions that can be tested in future experiments to verify our model and to distinguish the different dual regulation mechanisms (*H*_6_ versus *H*_7_). Our model also shows that the transient phosphorylation kinetics depend on the initial incubation process (premixing P1 or ATP with the enzyme), which can also be tested by future experiments. In particular, the phosphoryl transfer rates can be measured continuously (at least at multiple times) in the time window of 0–100 s. If the time dependence follows that of the blue (red) line shown in Fig 6, it would indicate that the underlying mechanism is *H*_7_ (*H*_6_). See S6 Appendix for details.

In general, understanding microscopic mechanisms in biological systems is challenging given the complexity of the underlying processes and the difficulty in measuring individual reactions. Here, we show that combining modeling of the dynamics of the whole reaction network with quantitative system level “input-output” measurements provides a powerful tool to address this challenge, as demonstrated here in the case of kinase CheA regulation. This systems-biology approach, which includes the development of a mechanistic network model based on key underlying biochemical reactions and searching the hypothesis space by fitting a large body of input-output data to the model, should be generally applicable to the study of other biological regulatory systems.

## Supporting information

S1 Appendix The full mathematical model. (PDF)

S2 Appendix Alternative models where receptors change the equilibrium properties of the enzyme. (PDF)

S3 AppendixSimplifying the model with $k_{\text{off}}^{\text{ATP}}$ and $k_{\text{off}}^{\text{P1}}$ regulation.(PDF)

S4 AppendixSimplifying the model with $k_{\text{off}}^{\text{ATP}}$ and $k_{f}^{P}$ regulation.(PDF)

S5 Appendix The effective Michaelis-Menten parameters from simulations of the model. (PDF)

S6 Appendix Model predictions for premixing enzyme and ATP. (PDF)
