# Supplementary material for: A dual regulation mechanism of histidine kinase CheA identified by combining network-dynamics modeling and system-level input-output data
Source: PLoS Comput Biol. 2018 Jul 2;14(7):e1006305. doi: 10.1371/journal.pcbi.1006305 (PMC6044545; doi:10.1371/journal.pcbi.1006305)
Supplement: S1 Appendix — (PDF) [file pcbi.1006305.s001.pdf]

Supplementary Material to
A dual regulation mechanism of histidine kinase CheA identified by combining network-dynamics modeling and system-level input-output data
Bernardo A. Mello, Wenlin Pan, Gerald L. Hazelbauer, and Yuhai Tu

Appendix 1

The full mathematical model

The concentrations of the nine enzyme configurations shown in Fig. 1 are described by the vector $\vec{E}$, defined as

$$\vec{E}(t) = \begin{bmatrix} [\mathrm{ATP}\cdot E\cdot\ \mathrm{P1}\ ](t) \\ [\mathrm{ATP}\cdot E\cdot\ \ \ \ ](t) \\ [\mathrm{ATP}\cdot E\cdot \mathrm{P1P}](t) \\ [\ \ \ \ \cdot E\cdot\ \mathrm{P1}\ ](t) \\ [\ \ \ \ \cdot E\cdot\ \ \ \ ](t) \\ [\ \ \ \ \cdot E\cdot \mathrm{P1P}](t) \\ [\mathrm{ADP}\cdot E\cdot\ \mathrm{P1}\ ](t) \\ [\mathrm{ADP}\cdot E\cdot\ \ \ \ ](t) \\ [\mathrm{ADP}\cdot E\cdot \mathrm{P1P}](t) \end{bmatrix}. \tag{A}$$

The total enzyme concentration, $[E]_{\mathrm{tot}}$, must be equal to the sum of the components of $\vec{E}$, which we define as the norm $\|\vec{E}(t)\|$. Enzyme conservation imposes

$$[E]_{\mathrm{tot}} = \|\vec{E}(t)\| = \mathrm{Constant}. \tag{B}$$

The experimental protocol results in the components of $\vec{E}$ been initially null, except the states $[\ \ \ \ \cdot E\cdot\ \ \ \ ]$ and $[\ \ \ \ \cdot E\cdot\ \mathrm{P1}\ ]$. We assume these configurations are in equilibrium at $t = 0$, with the values

$$[\ \ \ \ \cdot E\cdot\ \mathrm{P1}\ ](0) = f^{\mathrm{P1}}[E]_{\mathrm{tot}}, \tag{Ca}$$

$$[\ \ \ \ \cdot E\cdot\ \ \ \ ](0) = (1 - f^{\mathrm{P1}})[E]_{\mathrm{tot}}, \tag{Cb}$$

where f^{P1} is

$$f^{\mathrm{P1}} = \frac{[\mathrm{P1}]}{K_d^{\mathrm{P1}} + [\mathrm{P1}]}. \tag{D}$$

An equivalent approach was used in simulations with pre-mixing with ATP instead of P1.

The evolution of the enzyme vector is described by the equation

$$\frac{d\vec{E}}{dt} = A\vec{E}, \tag{E}$$

where A is the transition matrix

$$A = \begin{bmatrix} -k_{\text{off}}^{\text{ATP}} - k_{\text{off}}^{\text{P1}} - k_P^f & \omega^{\text{P1}} & 0 & \omega^{\text{ATP}} & 0 & 0 & 0 & 0 & k_r^P \\ k_{\text{off}}^{\text{P1}} & -k_{\text{off}}^{\text{ATP}} - \omega^{\text{P1}} & k_{\text{off}}^{\text{P1P}} & 0 & \omega^{\text{ATP}} & 0 & 0 & 0 & 0 \\ 0 & 0 & -k_{\text{off}}^{\text{ATP}} - k_{\text{off}}^{\text{P1P}} & 0 & 0 & \omega^{\text{ATP}} & 0 & 0 & 0 \\ k_{\text{off}}^{\text{ATP}} & 0 & 0 & -\omega^{\text{ATP}} - k_{\text{off}}^{\text{P1}} & \omega^{\text{P1}} & 0 & k_{\text{off}}^{\text{ADP}} & 0 & 0 \\ 0 & k_{\text{off}}^{\text{ATP}} & 0 & k_{\text{off}}^{\text{P1}} & -\omega^{\text{ATP}} - \omega^{\text{P1}} & k_{\text{off}}^{\text{P1P}} & 0 & k_{\text{off}}^{\text{ADP}} & 0 \\ 0 & 0 & k_{\text{off}}^{\text{ATP}} & 0 & 0 & -\omega^{\text{ATP}} - k_{\text{off}}^{\text{P1P}} & 0 & 0 & k_{\text{off}}^{\text{ADP}} \\ 0 & 0 & 0 & 0 & 0 & 0 & -k_{\text{off}}^{\text{ADP}} - k_{\text{off}}^{\text{P1}} & \omega^{\text{P1}} & 0 \\ 0 & 0 & 0 & 0 & 0 & 0 & k_{\text{off}}^{\text{P1}} & -k_{\text{off}}^{\text{ADP}} - \omega^{\text{P1}} & k_{\text{off}}^{\text{P1P}} \\ k_f^P & 0 & 0 & 0 & 0 & 0 & 0 & 0 & -k_{\text{off}}^{\text{ADP}} - k_{\text{off}}^{\text{P1P}} - k_r^P \end{bmatrix}. \tag{F}$$

Table A contains the definitions of the parameters used in this matrix. In the general case we should include in the above matrix the rate constants ω^{ADP} and ω^{P1P}, which were excluded because $[\text{ADP}] \approx [P1P] \approx 0$. Furthermore, the matrix A could be time-dependent, through the rates ω^{ATP} and ω^{P1} which depend on [ATP] and $[P1]$. Due to the minimum consumption of these substances, we assume the matrix to be constant.

The matrix A is the infinitesimal generator of the continuous time Markov process describing $\vec{E}$ evolution. All eigenvalues λ_i of such matrices are negative, except for of one which is null, $\lambda_0 = 0$.

By using the eigenvectors $\vec{E}_i$ of A, the initial state can be written as

$$\vec{E}(0) = \sum_{i=0}^{8} c_i \vec{E}_i, \tag{G}$$

where the coefficients c_i are determined by solving this linear system at time $t = 0$. With these coefficients, the state of the enzyme as function of time can be written as

$$\vec{E}(t) = c_0 \vec{E}_0 + \sum_{i=1}^{8} c_i \vec{E}_i e^{-t/\tau_i}, \tag{H}$$

where we used the timescale associated with each eigenvalue,

$$\tau_i = -\frac{1}{\lambda_i}. \tag{I}$$

We choose the index of the eigenvalues such that $\tau_1 > \tau_2 > \cdots > \tau_8$.

From Eq. (H) at $t \to \infty$ and Eq. (B), we conclude that

$$c_0 = \frac{[E]_{\text{tot}}}{\|E_0\|}. \tag{J}$$

The mean lifetimes of the transients, $\tau_1, \cdots, \tau_8$, are associated with the eigenvectors $\vec{E}_1, \cdots, \vec{E}_8$. The longest lasting transient is the one with the longest timescale, τ_1,

Reaction parameters

Symbol	Name	Definition
G^P	Ratio between k_f^P and k_r^P	Eq. (4)
$\bar{k}$	Mean reaction velocity per enzyme molecuel	Eq. (2)
k_{cat}^S	Catalytic rate constant of substrate S	Eq. (1)
$k_{\text{off}}^{\text{S}}$	Dissociation rate constant of substrate S	Eq. (3)
k_f^P	Phosphoryl group transfer rate	Eq. (4)
k_r^P	Reverse phosphoryl group transfer rate	Eq. (4)
K_d^S	Equilibrium dissociation constant of substrate S	Eq. (4)
K_m^S	MM dissociation constant of substrate S	Eq. (1)
v	Reaction rate	Eq. (1)
$\bar{v}$	Mean reaction rate	Eq. (2)
ω^S	*On* rate of substrate S	Eq. (3)

Concentrations

Symbol	Substance
$[E]$	Enzyme (P3P4P5)
$[E]_{\text{tot}}$	Total enzyme
$[\quad \cdot E \cdot \quad]$	Free enzyme
$[\text{A?P} \cdot E \cdot \quad]$	Enzyme bound to ATP or ADP
$[\quad \cdot E \cdot \text{P1?}]$	Enzyme bound to P1P or P1
$[\text{A?P} \cdot E \cdot \text{P1?}]$	Doubly bound enzyme
$[P1]$	Unphosphorilated
$[P1P]$	Phosphorilated P1
$[S]$	Substrate S

Table A. Symbols used in the paper. The role played by most of them are illustrated in Fig. 1.

which we will take as the *relaxation time*, $\tau \equiv \tau_1$. It means that the steady state is only reached when $t \gg \tau$. The steady state is the eigenvector $\vec{E}_0$ of the null eigenvalue.

The use of the SDS sample buffer in the experiments to stop the chemical reaction means that the measure of $[P1P]$ include the free and the bound proteins,

$$[\mathrm{P1P}_T] = [\mathrm{P1P}] + [\mathrm{ATP}{\cdot}E{\cdot}\mathrm{P1P}] + [\quad {\cdot}E{\cdot}\mathrm{P1P}] + [\mathrm{ADP}{\cdot}E{\cdot}\mathrm{P1P}]. \tag{K}$$

Alternatively, we can calculate $[\mathrm{P1P}_T]$ from the net P1 phosphorylation rate

$$v(t) = k_f^P[\mathrm{ATP}{\cdot}E{\cdot}\ \mathrm{P1}\ ] - k_r^P[\mathrm{ADP}{\cdot}E{\cdot}\mathrm{P1P}], \tag{L}$$

with $[\mathrm{ATP}{\cdot}E{\cdot}\ \mathrm{P1}\ ]$ and $[\mathrm{ADP}{\cdot}E{\cdot}\mathrm{P1P}]$ obtained from Eq. (H). We can now calculate the total phosphorylated P1 as

$$[\mathrm{P1P}_T](t) = \int_0^t v(t')dt'. \tag{M}$$

From this, we can write the mean phosphorylation rate as

$$\bar{v}(t) = \frac{1}{t}\int_0^t v(t')dt'. \tag{N}$$
