# Supplementary material for: A dual regulation mechanism of histidine kinase CheA identified by combining network-dynamics modeling and system-level input-output data
Source: PLoS Comput Biol. 2018 Jul 2;14(7):e1006305. doi: 10.1371/journal.pcbi.1006305 (PMC6044545; doi:10.1371/journal.pcbi.1006305)
Supplement: S2 Appendix — (PDF) [file pcbi.1006305.s002.pdf]

Supplementary Material to
A dual regulation mechanism of histidine kinase CheA identified by combining network-dynamics modeling and system-level input-output data
Bernardo A. Mello, Wenlin Pan, Gerald L. Hazelbauer, and Yuhai Tu

Appendix 2

Alternative models where receptors change the equilibrium properties of the enzyme

In cases in which receptor activity changes the equilibrium properties of the enzyme such as binding affinities, substrate binding can be different in the active and inactive state. Therefore, we use an equilibrium model for substrate binding that involves four states, E_l^a, for the substrate bound ($l = 1$) or not ($l = 0$) and for the active ($a = 1$) and inactive ($a = 0$) states. The free energy of each of these states may be written as

$$F_l^a = -a \ln \alpha - l \ln \frac{[S]}{K_d^a}, \tag{A}$$

where K_d^a represents the equilibrium dissociation constant of the active and inactive states, and α is

$$\alpha = \frac{\sigma}{1-\sigma} \tag{B}$$

which results in $0 \leq \sigma \leq 1$ and $0 \leq \alpha \leq \infty$.

The effective association and dissociation rate constants for this model are

$$k_{\text{on}} = \frac{k_{\text{on}}^0 + k_{\text{on}}^1 \alpha}{1+\alpha} \tag{Ca}$$

$$k_{\text{off}} = \frac{k_{\text{on}}^0 + k_{\text{on}}^1 \alpha}{1/K_d^0 + \alpha/K_d^1}, \tag{Cb}$$

where k_{on}^1 and k_{on}^0 are the association rate constants for the active and inactive state, and similarly for the dissociation rate constants. It is worth mentioning that expressions (C) require the steady state of the inactive/active switching, not the steady state of the substrate binding. These requirements are satisfied in the experimental conditions.

It is easy to see from Eq. (C) t hat when the receptor does not affect the equilibrium binding constant, i.e., $K_d^0 = K_d^1$, this general model reduces to the case where both the forward and backward rate constants are proportional to the same factor that is linear in σ, which is what we consider in most of this paper.

Similarly, the free energy of the phosphoryl transfer model is

$$F_p^a = -a \ln \beta + p \ln G^a, \tag{D}$$

where $p = 0$ is the state with ATP and P1 and $p = 1$ is the state with ADP and P1P, and the constant G^P has now different values in the active and inactive states. The value of β is

$$\beta = \frac{\sigma}{1-\sigma}, \tag{E}$$

and the effective rate constants are

$$k_f = \frac{k_f^0 + k_f^1 \beta}{1+\beta} \tag{Fa}$$

$$k_r = \frac{k_f^0 + k_f^1 \beta}{1/G^0 + \beta/G^1}. \tag{Fb}$$

The values of χ^2 obtained from the fitting with the singly regulated models using the effective rate constants (C) and (F) are shown in Fig. A.

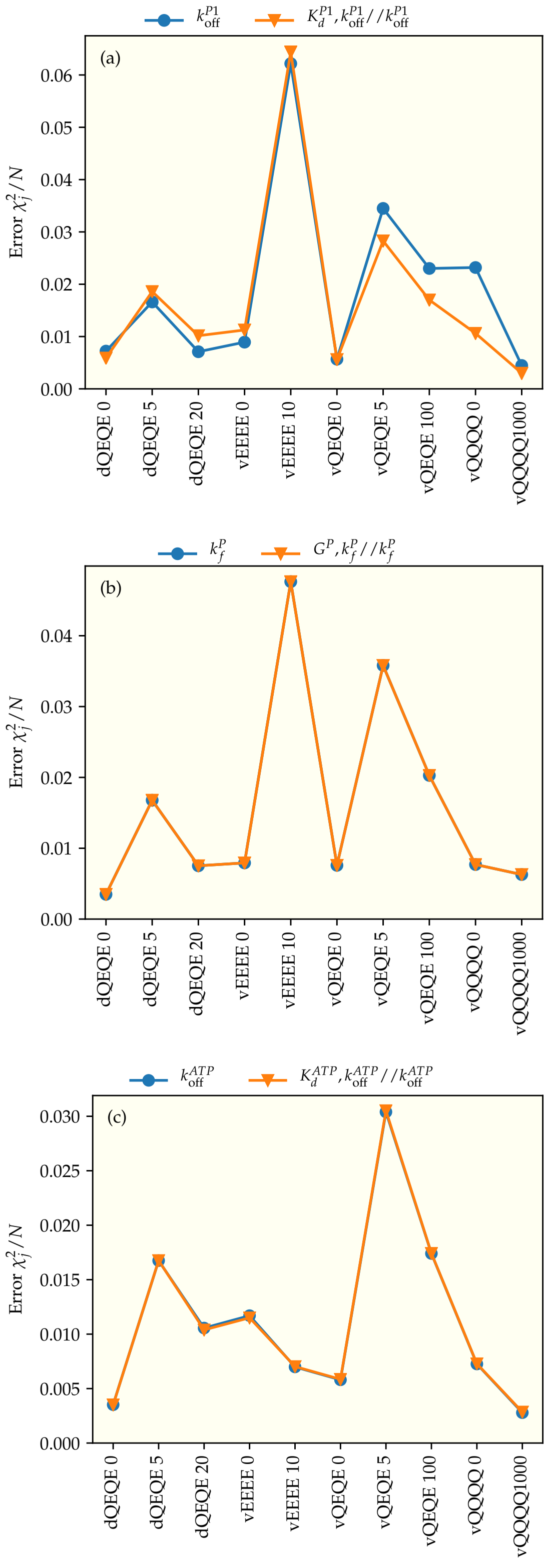

Fig A. Comparison of models with and without residual activities. Fitting errors in models with (orange) and without (blue) residual activities when the receptor activity regulates (a) P1 binding, (b) the phosphoryl transfer rate constant, (c) the ATP dissociation rate constant. The improvements of fitting by including residual activities are minimal. The orange and blue lines overlie each other almost perfectly.
