# Supplementary material for: A dual regulation mechanism of histidine kinase CheA identified by combining network-dynamics modeling and system-level input-output data
Source: PLoS Comput Biol. 2018 Jul 2;14(7):e1006305. doi: 10.1371/journal.pcbi.1006305 (PMC6044545; doi:10.1371/journal.pcbi.1006305)
Supplement: S3 Appendix — (PDF) [file pcbi.1006305.s003.pdf]

Supplementary Material to
A dual regulation mechanism of histidine kinase CheA identified by combining network-dynamics modeling and system-level input-output data
Bernardo A. Mello, Wenlin Pan, Gerald L. Hazelbauer, and Yuhai Tu

Appendix 3

Simplifying the model with $k_{\mathbf{off}}^{\mathbf{ATP}}$ and $k_{\mathbf{off}}^{\mathbf{P1}}$ regulation

A The five states model

The full model can be simplified by assuming that states connected by fast reactions and surrounded by slow reactions are in the steady state. The property $k_{\rm off}^{\rm P1} \gg k_{\rm off}^{\rm ATP}$ and $\omega^{\rm P1} \gg \omega^{\rm ATP}$ in Table 2 justify assuming the steady state in the binding of P1 to the enzyme.

In the five states model, we group together some pairs of states that differ only by the P1 binding site being empty in one state and occupied by P1 in the other. We use the following convention to represent these grouped states

$$[\quad \cdot E] = [\quad \cdot E\cdot \quad] + [\quad \cdot E\cdot\ \mathrm{P1}\ ], \tag{Aa}$$
$$[\mathrm{ADP}\cdot E] = [\mathrm{ADP}\cdot E\cdot \quad] + [\mathrm{ADP}\cdot E\cdot\ \mathrm{P1}\ ]. \tag{Ab}$$

In figure A(b) we see the states and the reactions of the five states model. The occupancy of the P1 binding site in the grouped states is given by Eq. (D) of S1 Appendix.

The much higher dissociation constant for P1P compared to ADP allow us to disregard the reaction ADP$\cdot E\cdot$P1P $\to$ $\cdot E\cdot$P1P. Because of the negligible concentration P1P and ADP the concentration of ATP$\cdot E\cdot$P1P and $\cdot E\cdot$P1P are also negligible. However we must keep [ADP$\cdot E\cdot$ P1P] and [ADP$\cdot E$], which results from the phosphoryl transfer reaction.

The relatioship ratio $k_{\rm off}^{\rm P1} \gg k_{\rm off}^{\rm ATP}$ make the five states model a good replacement for the full model in the experiment conditions. It is the starting points for further simplification.

B The complex four states model

If $k_f^P \gg k_{\rm off}^{\rm P1}$ we can define the state

$$[\mathrm{A^*P}\cdot E\cdot\ \mathrm{P1^*}] = [\mathrm{ATP}\cdot E\cdot\ \mathrm{P1}\ ] + [\mathrm{ADP}\cdot E\cdot \mathrm{P1P}]. \tag{B}$$

By assuming a steady state for the phosphoryl transfer reaction we can write [ADP$\cdot E\cdot$ P1P] $= f^P$[A*P$\cdot E\cdot$ P1*], with

$$f^P = \frac{1}{1+G^P}. \tag{C}$$

The resulting four states model, shown in Fig. A(c), can be used when $\sigma \ll k_f^P/\kappa_{\rm off}^{\rm P1}$ where $\kappa_{\rm off}^{\rm P1} \equiv k_{\rm off}^{\rm P1}/\sigma$.

C The simple four states model

If $k_{\rm off}^{\rm P1} \gg k_f^P$ we can assume steady state of P1 binding to ATP$\cdot E$ and define the state

$$[\mathrm{ATP}\cdot E] = [\mathrm{ATP}\cdot E\cdot \quad] + [\mathrm{ATP}\cdot E\cdot\ \mathrm{P1}\ ]. \tag{D}$$

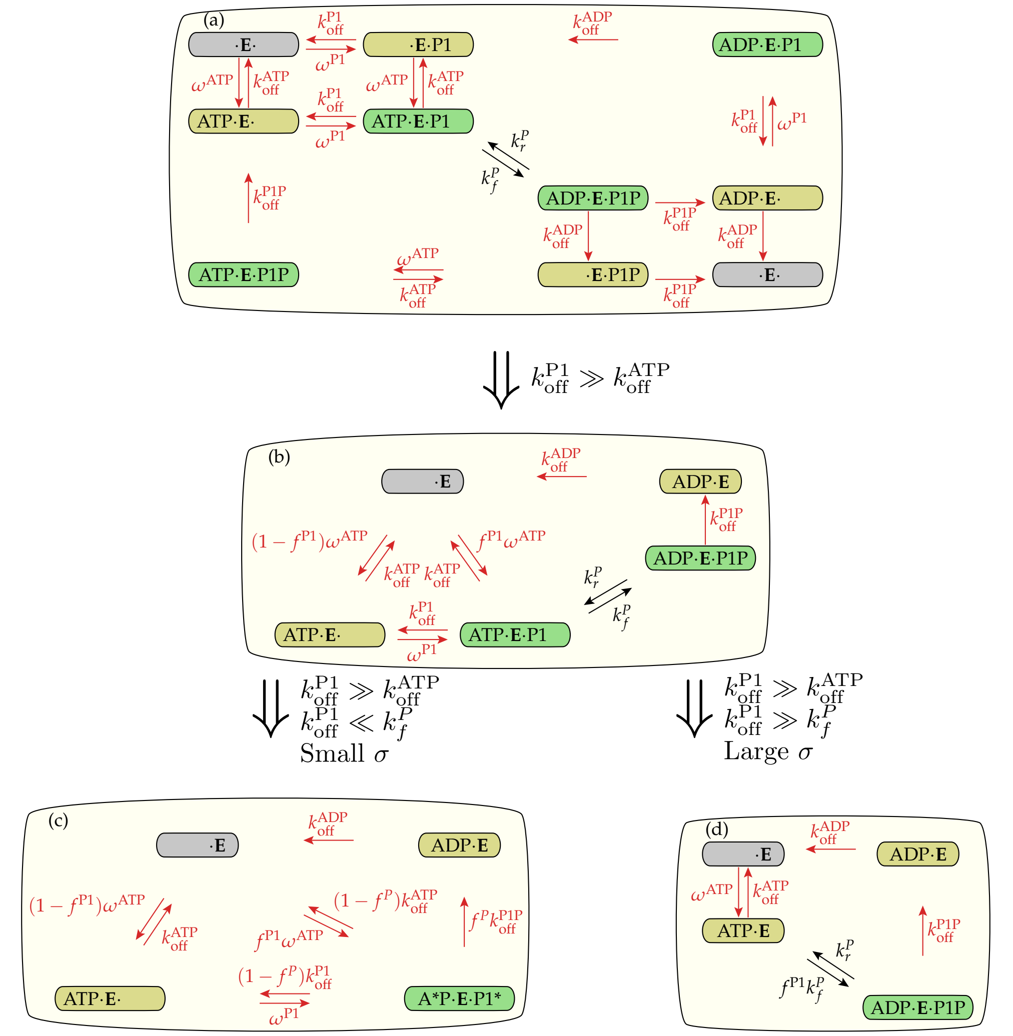

Fig A. Using parameters from Table 2 to simplify the enzymatic network of Fig. 1. (a) The same as Fig. 1 for the model with regulation of $k_{\text{off}}^{\text{ATP}}$ and $k_{\text{off}}^{\text{P1}}$, regulation represented by red arrows and rate constants. (b) The parameters allow disregarding some states and grouping together some states that differ only by the attachment of P1 . It results in the five states model, which describe the experimental data as well as the full-model. Four states models can be used when σ is small (c) or large (d).

This condition implies $\sigma \gg k_f^P / \kappa_{\text{off}}^{\text{P1}}$.

In figure A(d) we see the states and the reactions of the four states model for large σ. The phosphoryl transfer reaction is proportional to

$$[\text{ATP}\cdot E\cdot\ \text{P1}\ ] = f^{\text{P1}}[\text{ATP}\cdot E], \tag{E}$$

with f^{P1} given by Eq. (D) of S1 Appendix. Due to the high ratio $G^P = k_r^P / k_f^P$ shown in Table 2, the reverse phosphoryl transfer rate constant is much larger than the forward rate constant. For this reason we can't assume instantaneous detachment of P1P and the state [ADP$\cdot E\cdot$P1P] must be explicitly written. The value of $k_{\text{off}}^{\text{P1}}$ is irrelevant for this model, as long as it is large enough.

The simplicity of the four states model with large σ allows obtaining analytic expressions for the Michaelis-Menten parameters, as we do next. The mathematical model of Fig. A(d) requires a four-component enzyme vector,

$$\vec{E}(t) = \begin{bmatrix} [\text{ATP}\cdot E](t) \\ [\quad\ \cdot E](t) \\ [\text{ADP}\cdot E](t) \\ [\text{ADP}\cdot E\cdot \text{P1P}](t) \end{bmatrix}. \tag{F}$$

and the transition matrix

$$A = \begin{bmatrix} -k_{\text{off}}^{\text{ATP}} - f^{\text{P1}}k_f^P & \omega^{\text{ATP}} & 0 & k_r^P \\ k_{\text{off}}^{\text{ATP}} & -\omega^{\text{ATP}} & k_{\text{off}}^{\text{ADP}} & 0 \\ 0 & 0 & -k_{\text{off}}^{\text{ADP}} & k_{\text{off}}^{\text{P1P}} \\ f^{\text{P1}}k_f^P & 0 & 0 & -k_{\text{off}}^{\text{P1P}} - k_r^P \end{bmatrix}. \tag{G}$$

The null eigenvector of this matrix is

$$\vec{E}_0 = \begin{bmatrix} \dfrac{k_r^P + k_{\text{off}}^{\text{P1P}}}{f^{\text{P1}}k_f^P} \\ \dfrac{k_{\text{off}}^{\text{ATP}}}{\omega^{\text{ATP}}}\dfrac{k_r^P + k_{\text{off}}^{\text{P1P}}}{f^{\text{P1}}k_f^P} + \dfrac{k_{\text{off}}^{\text{P1P}}}{\omega^{\text{ATP}}} \\ \dfrac{k_{\text{off}}^{\text{P1P}}}{k_{\text{off}}^{\text{ADP}}} \\ 1 \end{bmatrix}, \tag{H}$$

which is also the steady state.

Instead of using Eq. (L) of S1 Appendix, we can calculate the steady state phosphorylation rate of the four states model as

$$v(t) = k_{\text{off}}^{\text{P1P}}[\text{ADP}\cdot E\cdot \text{P1P}](t) = k_{\text{off}}^{\text{P1P}}E^4(t), \tag{I}$$

where $E^4(t)$ is the fourth component of $\vec{E}(t)$ in Eq. (F). For the steady state, we can write

$$v = k_{\text{off}}^{\text{P1P}}c_0E_0^4 = k_{\text{off}}^{\text{P1P}}\frac{[E]_{\text{tot}}}{\|E_0\|}, \tag{J}$$

where we used Eq. (J) of S1 Appendix.

It is straightforward to show that Eq. (J) can be written as the Michaelis-Menten equation, Eq. (1), either as a function of [P1], with the constants

$$k_{\text{cat}}^{\text{P1}} = \left[\frac{1}{f^{\text{ATP}}}\left(\frac{G^P}{k_{\text{off}}^{\text{P1}}} + \frac{1}{k_f^P}\right) + \frac{1}{k^*}\right]^{-1}, \tag{Ka}$$

$$K_m^{\text{P1}} = \left(1 - \frac{k_{\text{cat}}^{\text{P1}}}{k^*}\right) K_d^{\text{P1}}, \tag{Kb}$$

$$\frac{1}{k^*} = \frac{1}{f^{\text{ATP}} k_{\text{off}}^{\text{ATP}}} + \frac{1}{k_{\text{off}}^{\text{P1}}},$$

or as a function of [ATP], with the constants

$$k_{\text{cat}}^{\text{ATP}} = \left(\frac{1}{f^{\text{P1}} k_f^P} + \frac{1}{k_{\text{off}}^{\text{ATP}}} + \frac{G^P/f^{\text{P1}} + 1}{k_{\text{off}}^{\text{P1}}}\right)^{-1}, \tag{Kc}$$

$$K_m^{\text{ATP}} = \left(1 - \frac{k_{\text{cat}}^{\text{ATP}}}{k_{\text{off}}^{\text{P1}}}\right) K_d^{\text{ATP}}. \tag{Kd}$$

In the above expressions we used the identity $k_{\text{off}}^{\text{ADP}} = k_{\text{off}}^{\text{ATP}}$ obtained from the fitting.

The concentration of ATP can affect $k_{\text{cat}}^{\text{P1}}$ and K_m^{P1} through the parameter f^{ATP} and the concentration of P1 can affect $k_{\text{cat}}^{\text{ATP}}$ and K_m^{ATP} through the parameter f^{P1}. The four Michaelis-Menten constants can be affected by the regulatory signal σ through k_f^P and $k_{\text{off}}^{\text{ATP}}$.
