# Supplementary material for: A dual regulation mechanism of histidine kinase CheA identified by combining network-dynamics modeling and system-level input-output data
Source: PLoS Comput Biol. 2018 Jul 2;14(7):e1006305. doi: 10.1371/journal.pcbi.1006305 (PMC6044545; doi:10.1371/journal.pcbi.1006305)
Supplement: S4 Appendix — (PDF) [file pcbi.1006305.s004.pdf]

Supplementary Material to
A dual regulation mechanism of histidine kinase CheA identified by combining network-dynamics modeling and system-level input-output data
Bernardo A. Mello, Wenlin Pan, Gerald L. Hazelbauer, and Yuhai Tu

Appendix 4
Simplifying the model with $k_{\mathbf{off}}^{\mathbf{ATP}}$ and k_f^P regulation

A The four states model

In Table A we can see that for this model $k_{\text{off}}^{\text{P1}} \gg k_{\text{off}}^{\text{ATP}}$ and $k_{\text{off}}^{\text{P1}} \gg k_f^P$, for all values of $\sigma < 1$. For this reason, the model shown in Fig. A(a) can be replaced by Fig. A(b). This model and the model of Fig. A(d) of S3 Appendix involve the same chemical reactions and share the same mathematical properties discussed in S3 Appendix and S4 Appendix.

B The three states model

Table A shows that if σ is sufficiently small, then we can assume $k_{\text{off}}^{\text{P1}} \gg G^P k_f^P$. In this case, the four states model of Fig. A(b) can be further simplified to A(c). This model requires a three-component enzyme vector,

$$\vec{E}(t) = \begin{bmatrix} [\text{ATP}\cdot E](t) \\ [\quad \cdot E](t) \\ [\text{ADP}\cdot E](t) \end{bmatrix}. \tag{A}$$

and the transition matrix

$$A = \begin{bmatrix} -k_{\text{off}}^{\text{ATP}} - f^{\text{P1}} k_f^P & \omega^{\text{ATP}} & 0 \\ k_{\text{off}}^{\text{ATP}} & -\omega^{\text{ATP}} & k_{\text{off}}^{\text{ADP}} \\ f^{\text{P1}} k_f^P & 0 & -k_{\text{off}}^{\text{ADP}} \end{bmatrix}. \tag{B}$$

The null eigenvector of this matrix is

$$\vec{E}_0 = \begin{bmatrix} 1 \\ \dfrac{k_{\text{off}}^{\text{ATP}} + f^{\text{P1}} k_f^P}{\omega^{\text{ATP}}} \\ \dfrac{f^{\text{P1}} k_f^P}{k_{\text{off}}^{\text{ADP}}} \end{bmatrix}, \tag{C}$$

which is also the steady state.

The phosphorylation rate may be obtained from

$$v(t) = k_{\text{off}}^{\text{ADP}} [\text{ADP}\cdot E](t) = k_{\text{off}}^{\text{ADP}} E^3(t), \tag{D}$$

where $E^3(t)$ is the third component of $\vec{E}(t)$ in Eq. (A). For the steady state, we can write

$$v = k_{\text{off}}^{\text{ADP}} c_0 E_0^3 = f^{\text{P1}} k_f^P \frac{[E]_{\text{tot}}}{\|E_0\|}, \tag{E}$$

$$v = [E]_{\text{tot}} \left(\frac{1}{f^{\text{ATP}} f^{\text{P1}} k_f^P} + \frac{1}{\omega^{\text{ATP}}} + \frac{1}{k_{\text{off}}^{\text{ADP}}} \right)^{-1}. \tag{F}$$

Reaction rates

$k_{\text{off}}^{\text{P1}}$	100 [49, ∞] s^{-1}	K_d^{P1}	209 [187, 236] μM
$k_{\text{off}}^{\text{ATP}}$	12.8 [9.2, 19.8] $\text{s}^{-1} \cdot \sigma$	$K_{d/D}^{\text{P1}}$	333 [289, 388] μM
k_f^P	21.7 [15.0, 33] $\text{s}^{-1} \cdot \sigma$	K_d^{ATP}	298 [273, 325] μM
G^P	10.1 [6.2, 14.6]		

Regulatory signal

State	[Asp]	σ	State	[Asp]	σ
dQEQE	20 μM	0.0068	vQEQE	100 μM	0.0116
	5 μM	0.123		5 μM	0.067
	0 μM	0.49		0 μM	0.31
vEEEE	10 μM	0.00030	vQQQQ	1000 μM	0.0176
	0 μM	0.00177		0 μM	1

Table A. Fitting parameters of model 6 of Table 1. Between brackets are the intervals of the reaction rates for a 1% increase on χ^2. The first letter of the state indicate the lipid bilayer into which the receptors are inserted: nano*d*isc or *v*esicle. The following four letters are the receptor's modification state.

By making $k_{\text{off}}^{\text{ATP}} = k_{\text{off}}^{\text{ADP}}$ we can write,

$$v = f^{\text{ATP}}[E]_{\text{tot}} \left(\frac{1}{f^{\text{P1}} k_f^P} + \frac{1}{k_{\text{off}}^{\text{ATP}}} \right)^{-1}. \tag{G}$$

which can also be obtained from Eq. (J) in S3 Appendix in the limit $\sigma = k_f^P \ll k_{\text{off}}^{\text{P1}}/G^P$. The Michaelis-Menten parameters for this equation are

$$k_{\text{cat}}^{\text{P1}} = f^{\text{ATP}} \frac{k_{\text{off}}^{\text{ATP}}}{k_{\text{off}}^{\text{ATP}} + k_f^P} k_f^P, \tag{Ha}$$

$$K_m^{\text{P1}} = \frac{k_{\text{off}}^{\text{ATP}}}{k_{\text{off}}^{\text{ATP}} + k_f^P} K_d^{\text{P1}}, \tag{Hb}$$

$$k_{\text{cat}}^{\text{ATP}} = \left(\frac{1}{f^{\text{P1}} k_f^P} + \frac{1}{k_{\text{off}}^{\text{ATP}}} \right)^{-1}, \tag{Hc}$$

$$K_m^{\text{ATP}} = K_d^{\text{ATP}}. \tag{Hd}$$

These functions are plotted as the black dotted lines in Fig. 7a-d with the values from Table 2, and perfectly match the exact model for $\sigma \ll k_{\text{off}}^{\text{P1}}/G^P$.

Due to the simplicity of the minimal model, the non-zero eigenvalues and eigenvectors with finite timescales are easily found when $k_{\text{off}}^{\text{ATP}} = k_{\text{off}}^{\text{ADP}}$,

$$\gamma_a = -k_{\text{off}}^{\text{ATP}} - \omega^{\text{ATP}}, \quad \vec{E}_a = \begin{bmatrix} 1 \\ f^{\text{P1}} k_f^P / \omega^{\text{ATP}} - 1 \\ -f^{\text{P1}} k_f^P / \omega^{\text{ATP}} \end{bmatrix} \tag{Ia}$$

$$\gamma_b = -k_{\text{off}}^{\text{ATP}} - f^{\text{P1}} k_f^P, \quad \vec{E}_b = \begin{bmatrix} 1 \\ 0 \\ -1 \end{bmatrix}. \tag{Ib}$$

It results in the following timescales

$$\tau_a = \frac{1}{\omega^{\text{ATP}}} f^{\text{ATP}}, \tag{Ja}$$

$$\tau_b = \frac{1}{k_{\text{off}}^{\text{ATP}} + f^{\text{P1}} k_f^P}. \tag{Jb}$$

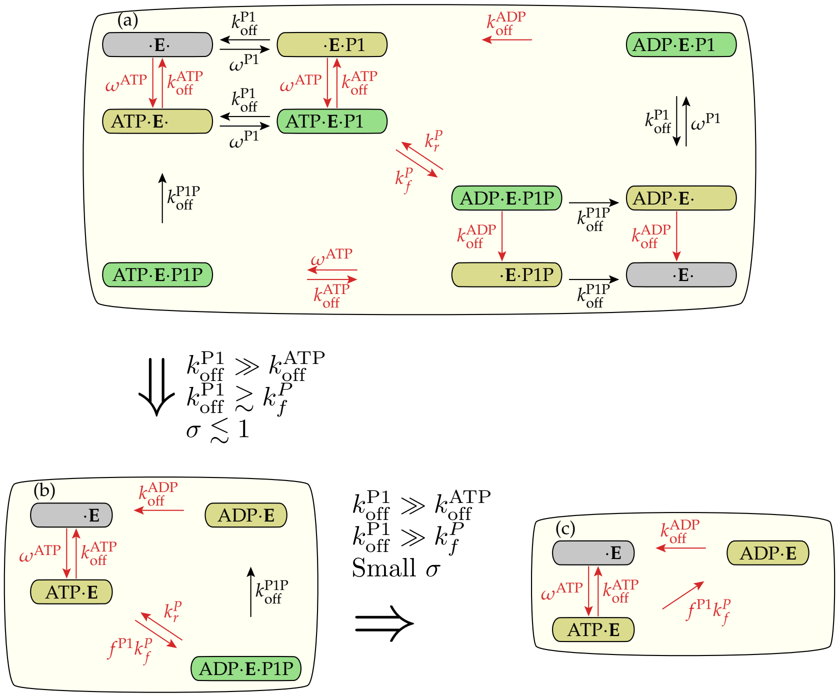

Fig A. Using parameters from Table A to simplify the enzymatic network of Fig. 1. (a) The same as Fig. 1 for the model with regulation of k_{off}^{ATP} and k_f^P, regulation represented by red arrows and rate constants. (b) By removing the non-essential reactions we can write the core model, which fits the data as well as the full-model. In the core model there are no states ATP·E·P1P or ·E·P1P and the small boxes represent the mix of enzymes with empty and occupied P1 binding sites. (c) A simpler model can be used if $\sigma \ll k_{off}^{P1}/G^P$. In this case, we can assume that the phosphoryl group is transferred from ATP to P1, but never from P1P to ADP.
