# Supplementary material for: A dual regulation mechanism of histidine kinase CheA identified by combining network-dynamics modeling and system-level input-output data
Source: PLoS Comput Biol. 2018 Jul 2;14(7):e1006305. doi: 10.1371/journal.pcbi.1006305 (PMC6044545; doi:10.1371/journal.pcbi.1006305)
Supplement: S5 Appendix — (PDF) [file pcbi.1006305.s005.pdf]

Supplementary Material to
A dual regulation mechanism of histidine kinase CheA identified by combining network-dynamics modeling and system-level input-output data
Bernardo A. Mello, Wenlin Pan, Gerald L. Hazelbauer, and Yuhai Tu

Appendix 5

The effective Michaelis-Menten parameters from simulations of the model

Figure A illustrate the dependence of the Michaelis-Menter parameters on the regulatory signal and substrate concentration. The simulations were performed for the same concentrations of the substrate that was held constant in the experiments, as indicated in the legends of Fig. 4. The Michaelis-Menten equation was used to obtain k_{cat}^{S} and K_m^S for the variable substrate.

The values of σ were not measured in the experiments. For this reason, it is not possible to compare the experimental results directly with Fig. A(a-d). To allow this comparison, we combined the points with the same σ from the curves of $k_{\mathrm{cat}}^{\mathrm{P1}}$ and K_m^{P1} to create the curve of $k_{\mathrm{cat}}^{\mathrm{P1}} \times K_m^{\mathrm{P1}}$ of Fig. A(e). The same procedure was used to create Fig. A(f).

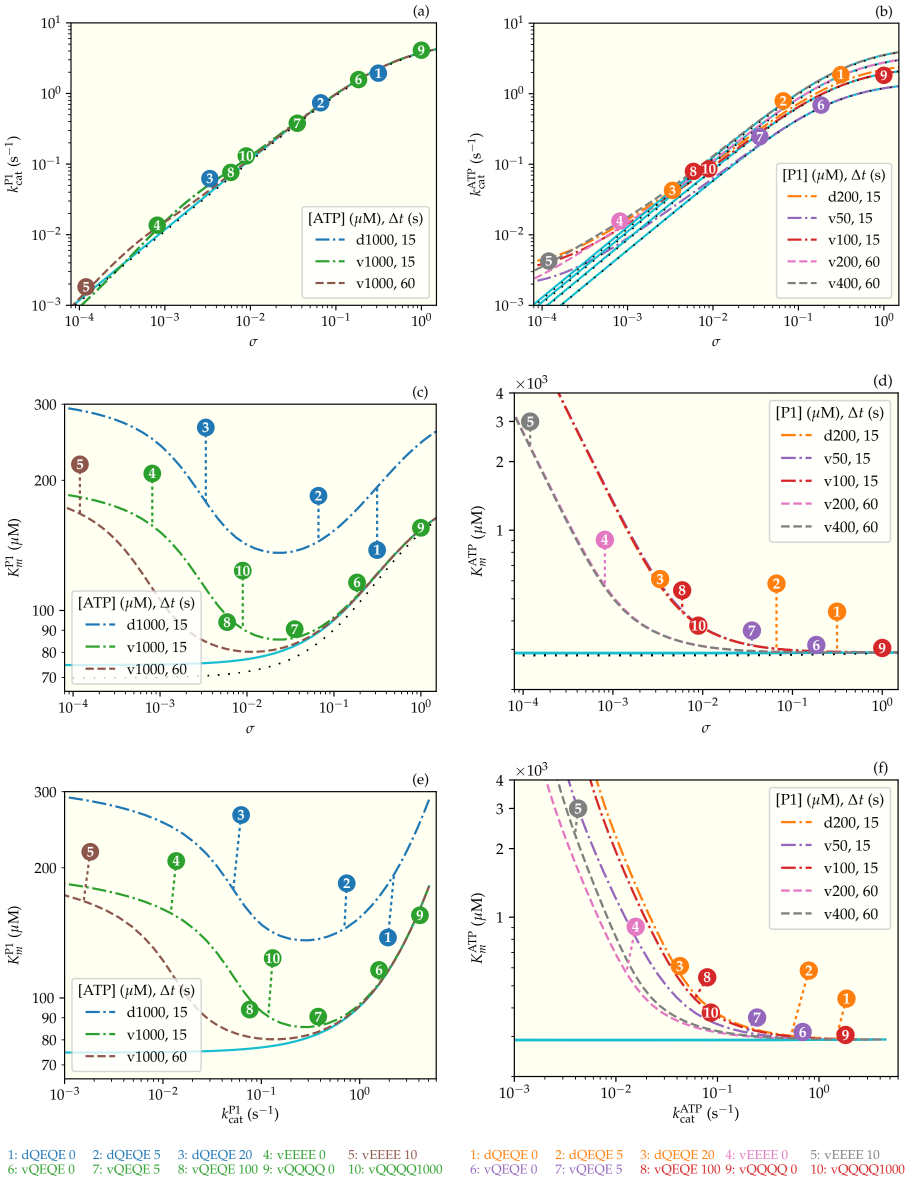

Fig A. Dependence of K_d^S and k_{cat}^S on σ. The curves show k_{cat}^S and K_m^S of the Michaelis-Menten equation, Eq. (1), for the phosphorylated rate predicted by the enzymatic reaction model. The parameters are calculated by fitting to curves similar to the continuous lines of Fig. 4. The simulations with $\Delta t = \infty$ (steady states) are the continuous lines. Simulations for the regulatory signal σ ranging from 10^{-3} to 10^2 were used to generate the curves of K_m^S and k_{cat}^S as functions of σ of plots (a), (b), (c), and (d).
