# Supplementary material for: A dual regulation mechanism of histidine kinase CheA identified by combining network-dynamics modeling and system-level input-output data
Source: PLoS Comput Biol. 2018 Jul 2;14(7):e1006305. doi: 10.1371/journal.pcbi.1006305 (PMC6044545; doi:10.1371/journal.pcbi.1006305)
Supplement: S6 Appendix — (PDF) [file pcbi.1006305.s006.pdf]

Supplementary Material to
A dual regulation mechanism of histidine kinase CheA identified by combining network-dynamics modeling and system-level input-output data
Bernardo A. Mello, Wenlin Pan, Gerald L. Hazelbauer, and Yuhai Tu

Appendix 6

Model predictions for premixing enzyme and ATP

Regulations of $k_{\mathrm{off}}^{\mathrm{P1P}}$, present in one model and absent in the other, can explain the differences caused by incubation (premixing), shown in Fig. A. The low concentration of P1 and the high concentration of ATP make P1 binding the limiting reaction at the onset of the experiment. For this reason, changes in the regulatory signal will have distinct effects on the phosphorylation rate measured during the transient, depending whether or not P1 binding is regulated.

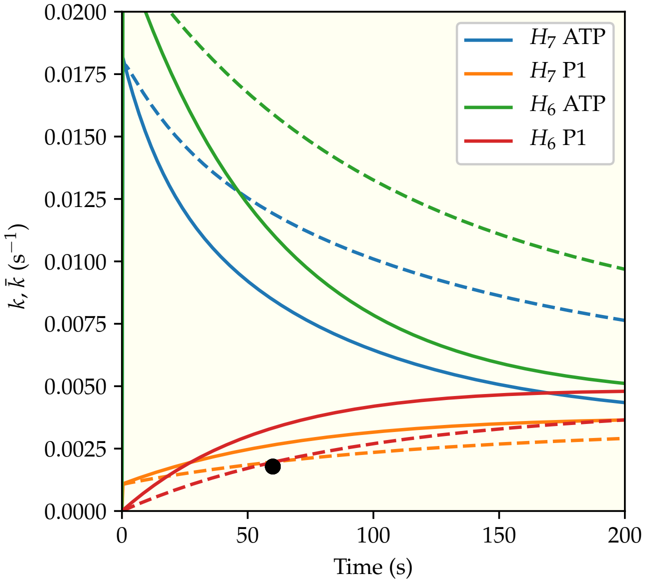

Fig A. The predicted time dependence of the apparent phosphoryl transfer rates for the two different dual-regulation mechanisms (H_6 and H_7) and incubation with ATP or P1, represented with different colors indicated in the legend. The system studied here contains the least active receptors (EEEE) in membrane vesicles with [Asp]=10 μM, [ATP]=10^3 μM, and [P1]=25 μM, which reproduces the conditions of the leftmost point of the lower curve in Fig. 4(c). The solid and the dashed lines correspond to the instantaneous (k) and the average ($\bar{k}$) phosphorylation rate constant respectively. The black circle is the experimental data point from [17].

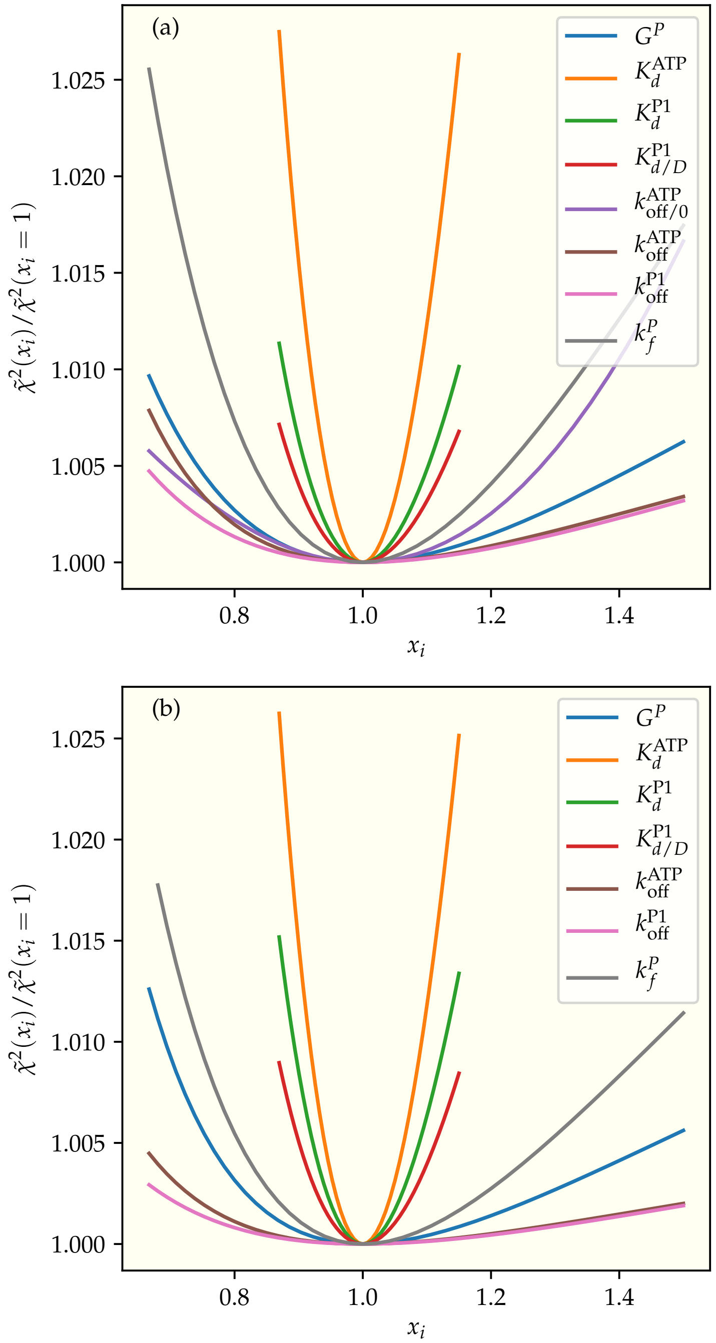

Fig B. Improving the fitting by including one parameter. The vertical axis is the value of χ^2 when the parameter in the legend is kept constant, at the value indicated in the horizontal axis, and the other are fitted to the data. In Table 1, models 8 and 7, respectively (a) and (b) above, differs only by one the addition of parameter $k_{\mathrm{off/0}}^{\mathrm{ATP}}$ in model 8, accounting for a different dissociation constants for nanodiscs. Values on both axes are relative to the values at the minimum of χ^2.
